# The role of lymphangiogenesis in cardiovascular diseases and heart transplantation

**DOI:** 10.1007/s10741-021-10188-5

**Published:** 2021-11-04

**Authors:** Rui-Cheng Ji

**Affiliations:** Faculty of Welfare and Health Science, Oita University, Oita, 870-1192 Japan

**Keywords:** Lymphangiogenesis, VEGF-C/VEGFR-3, Cardiovascular diseases, Atherosclerosis, Myocardial infarction, Heart transplantation

## Abstract

Cardiac lymphangiogenesis plays an important physiological role in the regulation of interstitial fluid homeostasis, inflammatory, and immune responses. Impaired or excessive cardiac lymphatic remodeling and insufficient lymph drainage have been implicated in several cardiovascular diseases including atherosclerosis and myocardial infarction (MI). Although the molecular mechanisms underlying the regulation of functional lymphatics are not fully understood, the interplay between lymphangiogenesis and immune regulation has recently been explored in relation to the initiation and development of these diseases. In this field, experimental therapeutic strategies targeting lymphangiogenesis have shown promise by reducing myocardial inflammation, edema and fibrosis, and improving cardiac function. On the other hand, however, whether lymphangiogenesis is beneficial or detrimental to cardiac transplant survival remains controversial. In the light of recent evidence, cardiac lymphangiogenesis, a thriving and challenging field has been summarized and discussed, which may improve our knowledge in the pathogenesis of cardiovascular diseases and transplant biology.

## Introduction

The lymphatic system is required for the maintenance of tissue homeostasis by returning extracellular fluids, macromolecules, and immune cells from peripheral tissues to the draining lymph nodes (LNs), and finally back to the blood circulation. Lymphangiogenesis is involved in many pathological processes, e.g., inflammation, wound healing, lymphedema, and tumor metastasis, acting as a channel for transporting signaling molecules and immune cells between injured tissues and regional LNs [1–5]. In recent years, great advances in cardiac lymphangiogenesis have been made to elucidate the molecular and pathological mechanisms using both the zebrafish and mouse models, which provide an experiment platform to investigate lymphatic-related cardiovascular diseases [6–8]. Cardiac studies based on state-of-the-art imaging technologies and genetic models have focused on lymphatic endothelial cells (LECs) in more detail, revealing tissue-specific heterogeneity in origin, function, and response to injury [9–11]. A recent study has highlighted the importance of LECs and LEC-specific matrix molecule, REELIN during heart growth and repair, and provided valuable ideas about possible paths to improve cardiac regeneration and cardio-protection in mammals [12]. The important findings suggest that the use of REELIN could be a valuable therapeutic approach to improve cardiac function in humans [12]. Moreover, a growing body of evidence indicates that lymphangiogenesis is an active participant in the pathogenesis of atherosclerosis and myocardial infarction (MI) [13–15]. Vascular endothelial growth factor C (VEGF-C) and its receptor VEGFR-3 facilitate lymphangiogenesis and lymphatic function, which may provide a pathway for inflammatory cell efflux, favoring the resolution of cardiac edema and wound healing within the injured heart [16].

Targeting lymphangiogenesis as a potential strategy to prevent or treat some cardiovascular diseases has become a remarkable research topic in recent years. However, insufficient or maladaptive cardiac lymphatic remodeling has been considered to cause impaired lymph transport capacity and accumulation of protein-rich fluid, contributing to chronic myocardial inflammation and edema, which triggers development of interstitial fibrosis [17]. In this field, it remains to be further clarified: (1) how cardiac lymphangiogenesis affects the healing process of the injured heart like MI; (2) why the insufficient or defective lymphangiogenesis contributes to myocardial edema and fibrosis; (3) how the complex interplay between lymphangiogenesis and immune response modulates cardiac function and homeostasis. Finally, it remains unclear and controversial whether and how increased lymphangiogenesis improves lymph drainage function and affects transplant rejection. The alloimmune response resulting from the interaction of innate and adaptive immunity may be detrimental to cardiac allografts and heart transplant recipients [18, 19]. For this reason, VEGFR-3 inhibition could be used as lymphatic-targeted immunomodulatory therapy to prevent acute and chronic rejection in cardiac allografts [20]. Reduction of lymphangiogenesis has been reported to protect allogenic heart transplants and beyond [19, 21]. Therefore, it is important to understand the diversity and complexity of the interplay between lymphangiogenesis and transplantation immunology.

In this context, the present review has mainly summarized the advancement of lymphangiogenesis in prevalent cardiovascular diseases and beyond, based on animal disease models and clinicopathological studies, which may provide information about the outcomes of future clinical trials in this field.

## Morphological and pathological characteristics of the cardiac lymphatics

**Fig. 1:**
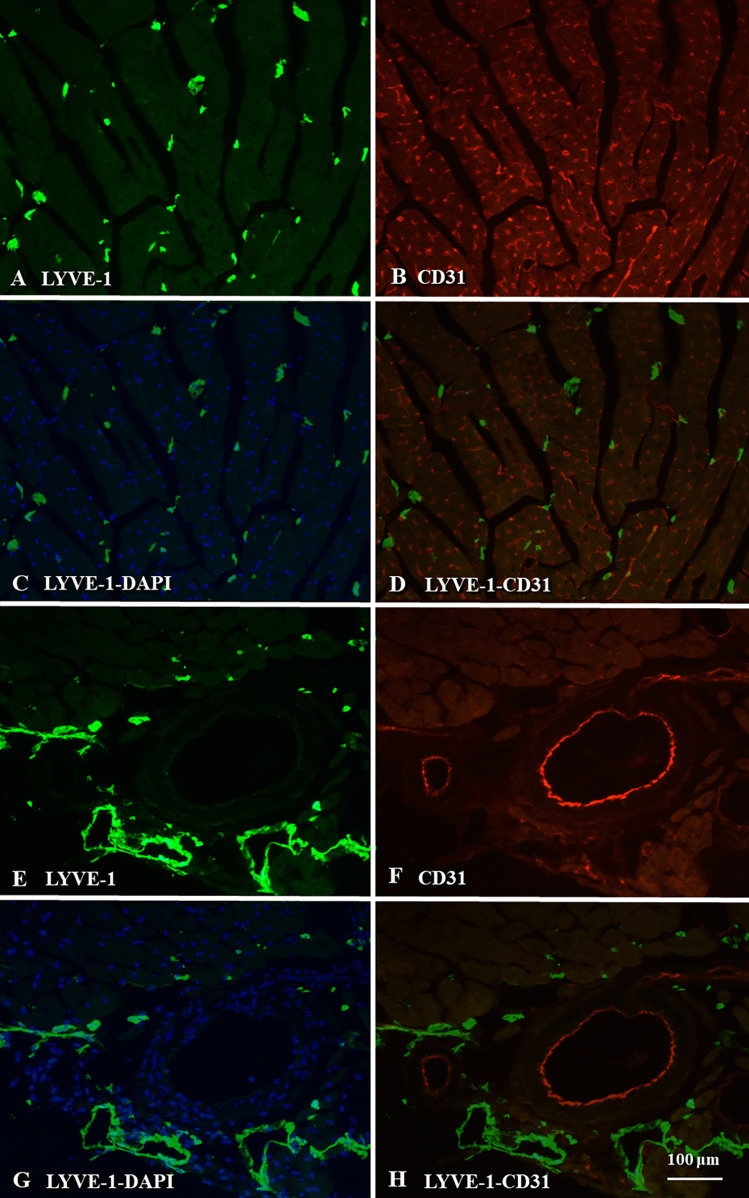
Lymphatics of cardiac muscles in C57BL/6 J mice. Immunofluorescence staining with the lymphatic vessel endothelial hyaluronan receptor 1 (LYVE-1, green) for lymphatics, CD31 (red) for blood vessels, and 4′,6-diamidino-2-phenylindole dihydrochloride (DAPI, blue) for nuclear counterstaining in the cardiac muscle (**A** ~ **H**). Initial lymphatics are mainly surrounding myocardial fibers, and fewer than the blood capillaries (**A** ~ **D**). Moreover, the relationship between irregular collecting lymphatics and blood vessels is clearly identified in perimysial connective tissues of the heart (**E** ~ **H**)

**Fig. 2:**
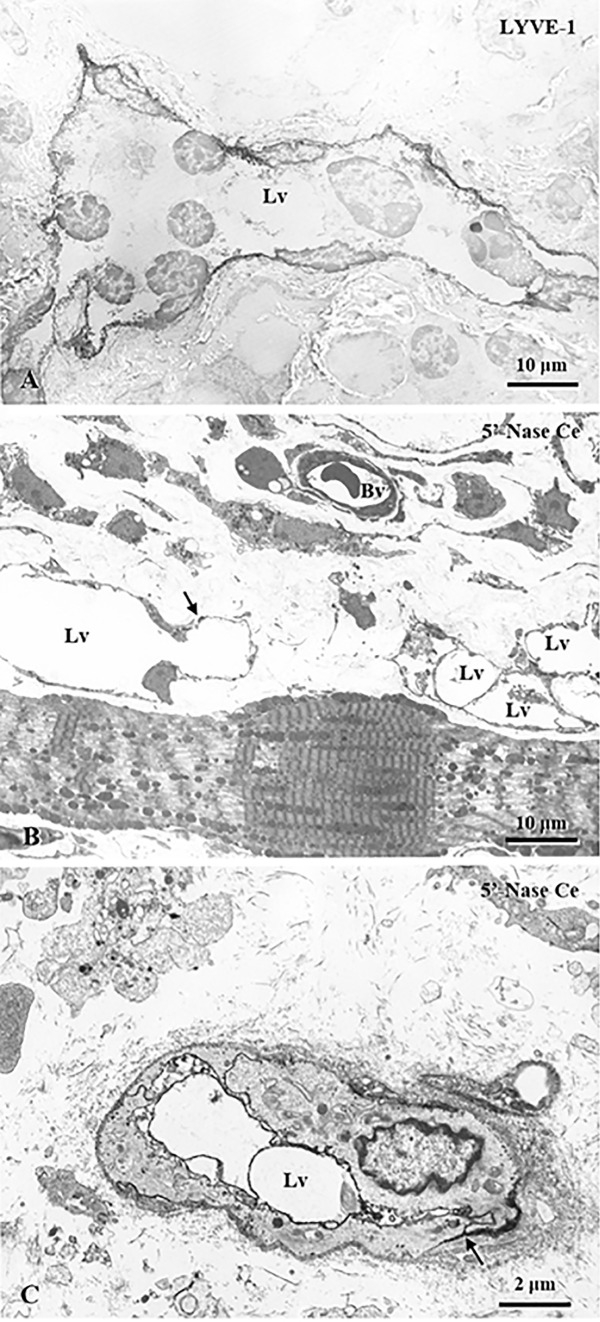
Transmission electron micrographs (TEM) of the intermuscular lymphangiomas in the diaphragm of BALB/c mice. Near abdominal muscular fibers of the diaphragm, the endothelial cells are decorated with LYVE-1 and 5′-nucleotidase (5′-Nase)-cerium reactive precipitates (**A**–**C**) to confirm new formation of initial lymphatics. The developmental lymphatic structures have a definite endothelial lining with simple intercellular junctions (**B**, **C**, arrows). Lv lymphatic vessels, Bv blood vessels

Cardiac lymphatics play an important role in maintaining tissue fluid balance and immune surveillance, which are implicated in cardiovascular diseases. The cardiac lymphatic networks are generally distributed in all the three layers, subendocardial, myocardial, and subepicardial, in which the subendocardial layer contains fewer initial lymphatics as compared to subepicardium in mammalian including human [22]. Moreover, intramyocardial lymphatics in mice are fewer in comparison with an abundant subepicardial lymphatic network [23], and cardiac lymph flow begins from small subendocardial lymphatics and continues through myocardium into subepicardial capillaries that converge into larger collecting lymphatics [24]. The intramyocardial lymphatics in rats also drain toward the subepicardial network composed of blind-ended capillaries, larger pre-collecting, and valved collecting lymphatics [25]. Generally, initial lymphatics are mainly surrounding myocardial fibers but fewer than the blood capillaries, whereas irregular collecting lymphatics can be clearly identified in perimysial connective tissues of the heart accompanying blood vessels (Fig. 1). In addition, the newly formed lymphatics have a definite endothelial lining with simple intercellular junctions (Fig. 2). In peripheral tissues, the initial lymphatics are consisting of a single layer of LECs, with overlapping, interdigitating and open intercellular junctions, but the collecting lymphatics with intraluminal valves possess a smooth muscle cell layer and basement membrane [26].

The embryological origin and development of the cardiac lymphatics have provided some important implications on how to address cardiovascular fluid homeostasis, injury-induced inflammation, and healing process. The study of embryonic lymphangiogenesis in mice has indicated that the cardiac LECs expressing VEGFR-3, prospero homeobox-1 (*Prox-1*), and lymphatic vessel endothelial hyaluronan receptor-1 (LYVE-1) have a heterogeneous cellular origin derived from the common cardinal vein and second heart field. The mouse cardiac lymphatics begin to develop from around mid-gestation, at approximately embryonic day 12.5 (E12.5) and become evident at around E14.5. As development progresses, an extensive lymphatic network expands over both the dorsal and ventral surfaces, and into the myocardium to form a lymphatic network during late embryonic and postnatal stages [7, 27–29]. In most mammalian species, initial lymphatics cover the subendocardium and myocardium, and atrioventricular valves [22]. Increased lymphatic network in cardiac valves, interestingly, may contribute to resolving infective endocarditis in patients [30].

Studies on animal models by blocking cardiac lymph flow or accelerating plaque formation are available to understand the importance of lymphangiogenesis in MI and atherosclerosis [11, 31]. Lymphangiogenesis has provided a route for improving clearance of immune cells and cardiac repair process in MI. The adult heart undergoes significant lymphangiogenesis following initiation of a developmental program in response to ischemic injury. In comparison with a remarkable increase in the branching of surface VEGFR-3^+^ lymphatics, and alignment of *Prox-1*^+^ lymphatic sprouting with Emcn^+^ veins at day 7 following injury, a prominent spatiotemporal change in the lymphatic response occurs from days 7 to 35 post-MI [7]. Functional post-embryonic VEGF-C signaling in adult zebrafish is critical for the outgrowth of cardiac lymphatics along the ventricular arteries. The presence of necrotic tissue and scar formation in a cardiac cryoinjury model promotes ventricular lymphangiogenesis due to increased VEGF-C levels [8].

During heart growth and repair, the interplay of LECs and some other signal molecules, e.g., the integrin-linked kinase (ILK) has provided more information about possible pathways for linking to cardiovascular development and diseases. ILK via controlling the interaction of VEGFR-3 with integrin-β1 regulates LEC proliferation and prevents lymphatic overgrowth during mouse embryonic development. ILK knockout mice have shown to increase cardiac lymphangiogenesis in post-MI recovery period [32]. In a mouse model with permanent coronary artery ligation, the phenotypically heterogeneous podoplanin-expressing cell populations are likely involved in lymphatic growth and fibrogenic response to infarcted myocardium [33].

Cardiac lymphatics may undergo significant remodeling including deformed initial and collecting lymphatics, closely relating to the pathological process of atherosclerosis, MI, and heart transplantation. Therefore, the possible involvement of lymphangiogenesis will be emphasized in the following sections in order to provide the necessary information for future potential therapeutic application.

## Lymphangiogenesis in cardiovascular diseases

### Atherosclerosis

Atherosclerosis is a progressive disease characterized by formation of plaques on the inner walls of arteries consisting of extracellular lipid, cholesterol crystals, inflammatory cells, and necrotic debris [34]. The accumulation of arterial cholesterol is the most common phenotype in atherosclerosis [35]. Lymphatic dysfunction in the arterial wall, either duo to surgical ligation or removal of collecting lymphatics, and even congenital lymphatic insufficiency or malformation will greatly impair reverse cholesterol transport (RCT) and stimulate atherosclerotic lesion formation [36, 37]. RCT is defined as the process for the removal and transport of excess cholesterol from peripheral tissues to the liver via plasma for excretion. RCT is initiated from cellular cholesterol efflux facilitated by lipid-free apolipoprotein A1 (ApoA1) or other high-density lipoprotein (HDL) particles within the interstitial space where extracellular cholesterol is picked up and transported through the lymphatics before entering the circulation [38]. In human and mouse atherosclerotic plaques of coronary and carotid arteries, a functional lymphatic network is present in the adventitia, where lymphatic expansion and increased lymphatic vessel density (LVD) have been found to be consistent with the lesion severity [30, 39, 40]. Induction of lymphangiogenesis by injection of VEGF-C, the ligand for VEGFR-3, into the mouse footpad leads to increased RCT and decreased cholesterol content in the plaque [41]. Moreover, stimulating lymphangiogenesis with VEGF-C_C152S_ promotes early rescue of lymphatic function for limiting macrophage accumulation, and increasing immune cell migration through the lymphatics, and consequently restraining plaque development in low density lipoprotein (LDL) receptor knockout (Ldlr^−/−^) mice [42]. Macrophage RCT from advanced atherosclerotic lesions in Apoe^–/–^ mice can be impaired by anti-VEGFR-3 mAb that attenuates lymphatic growth and induces a defect in the absorption of lymph from peripheral tissues [43].

Recently, particular attention has been paid to RCT and atherosclerosis development, as well as their relation with arterial lymphatic drainage. However, the role of organ-specific lymphatics in modulating HDL transport and composition is incompletely understood [44]. Especially, the lymphatic origin and flow vary among tissues, e.g., lymphatic drainage in the skin, intestine, liver and peritoneal cavity, which may further reflect the importance of lymphatics in RCT and atherosclerosis. Lipid loading of macrophages and their transformation into foam cells is considered a decisive process in the formation and development of atherosclerotic lesions within the peritoneal cavity [45]. Preferential uptake into the lymphatics is highly dependent on the physicochemical properties of the particles, including size, molecular weight, surface charge, and lipophilicity [46]. Initial lymphatics draining the peritoneal submesothelial layer have favorable absorption of lipophilic and unionized compounds [47]. The study by using nonfasted rats has demonstrated that the lipoproteins in lymph are organ specific in composition, and the intestine and liver appear to be the main source of HDL that is modified during transport from the mesenteric and hepatic lymphatics to the thoracic duct [44]. The lymph lipoprotein remodeling may indicate an alteration of its biological properties and potentially impact on the development of atherosclerosis. Moreover, a substantial fraction of ApoA1 is continually found in the skin, the largest organ of the body, and thus the skin is a key player in the HDL cycle regardless of how lipoproteins are transported from arteries [36]. Indeed, skin lymphatics participate critically in RCT from peripheral tissues, where macrophage RCT is especially relevant. The experiments have shown that genetic ablation of lymphatics in Chy mice carrying 1 mutant allele of VEGFR-3 disrupts RCT from the skin, whereas interestingly, anti-VEGFR-3 mAb treatment does not lead to impaired dermal lymphatic transport [43], suggesting that lymphatic transport function differs according to the nature of intervention of lymphangiogenesis.

Lymphangiogenesis is thought to be involved in the development and pathogenesis of lymphatic-related disorders as a consequence of recruitment of activated immune cells, e.g., macrophages, T and B cells, mast cells, and dendritic cells (DCs). These cells regulate inflammation resolution and subsequent repair process by secreting prolymphangiogenic factors and anti-inflammatory mediators [48–52]. In atherosclerosis, lymphangiogenic areas are morphologically rich in scattered calcium deposits and cholesterol crystals and characterized by low or no cellular infiltrates [30]. Insufficient lymphangiogenesis might contribute to formation of atherosclerotic lesions in large arteries owing to accumulation of lipid and activated immune cells [53]. Continuous recruitment of monocytes into plaques, in particularly, drives the progression of the chronic inflammatory condition that is sustained, at least in part, by undesirable immunity against cholesterol-associated apolipoproteins [54–56]. A recent study has shown that CD36 deletion in mice and its siRNA-mediated knockdown in LECs can prevent oxidized LDL (oxLDL)-induced inhibition of lymphangiogenesis, which suggests that blockade of CD36 signaling of human LECs may promote adventitial lymphangiogenesis, leading to increased removal of arterial cholesterol and delayed atherosclerotic development [57]. In another murine model of atherosclerosis, either blockage of lymph drainage or inhibition of VEGFR-3-dependent lymphangiogenesis will result in increased CD3^+^ T cell density in plaque intima and adventitia, and deteriorate atherosclerosis, indicating that lymphangiogenesis is responsible for T cell drainage from the lesion [40]. Presumably, adventitial lymphatics are much required to inhibit the development of atherosclerosis and promote plaque regression. Lymphangiogenesis is favorable for facilitating cholesterol clearance and delaying atherosclerotic plaque formation depending on various cellular and molecular components, e.g., macrophages, the leucine-rich repeat-containing G protein-coupled receptor 4 (LGR4), and the chemokine (C-X-C motif) ligand 12/receptor 4 (CXCL12/CXCR4) (Table 1). Concerning protection against atherosclerosis, it should be considered how lymphangiogenesis targeting the arterial wall reduces lipid accumulation and subsequent inflammatory response, especially in humans.

**Table 1:** Lymphangiogenesis in atherosclerosis

Animals/human	Main targets (cytokine/chemokine/growth factor) and others	Lymphangiogenic properties and lymphatic features	References
Human tissue ApoE^−/−^ mice	Plaque tissue (carotid artery) AAV-hVEGFR3-Ig, i.v Transfection with CXCL12-specific siRNA	The dissection of LNs and lymphatics deteriorates atherosclerotic development by promoting T cell accumulation Inhibition of lymphangiogenesis increases plaque T cell content CXCL12/CXCR4 axis is associated with mouse plaque lymphangiogenesis and human plaque LVD	[40]
*ApoE*^*−/−*^, *Ldlr*^*−/−*^ mice SR-BI^+*/−*^ mice	Lymphatic functional assessment (Evans blue dye) LYVE-1, α-SMA, podoplanin	Lymphatic structure and function are restored by reducing hypercholesterolemia Lymph drainage is required for SR-BI-mediated HDL cholesterol transport VEGF-C may improve lymph transport and attenuate peripheral lipid accumulation	[41]
Ldlr^−/−^ mice	VEGF-C _152S,_ i.p VEGFR-3, FOXC-2	VEGF-C limits plaque formation for improving lymph transport and inflammatory cell migration via upregulated VEGFR-3 and FOXC-2 expression in LECs	[42]
*Apoa1* transgenic mice Chy mutant mice	Lymphatic separation Anti-VEGFR3 mAb, i.p podoplanin, LYVE-1	Surgical ablation of lymphatics blocks RCT without impairing cholesterol efflux from macrophages Genetic ablation of lymphatics disrupts RCT RCT from atherosclerotic aorta is impaired by anti-VEGFR-3 mAb inhibiting lymphatic growth	[43]
Human tissue CD36^*−/−*^ mice	Atherosclerotic tissue (aortic and coronary artery) In vivo lymphangiogenesis assay LYVE-1, eNOS, AKT, CD36, oxLDL, β-tubulin	oxLDL levels are increased in human atherosclerotic arteries oxLDL inhibits lymphangiogenesis and expression of AKT and eNOS in LECs CD36 silencing rescues inhibitory effects of oxLDL on cell cycle	[57]
*Apoe*^*−/−*^ mice *Ldlr*^*−/−*^ mice	Aortic lymphatic ligation VEGFC-_156S_, i.p LYVE-1, CD68, VEGFR-3, *Prox-1*, podoplanin, CD11c, IL-1β, IL-6, TNF-α	Expansion of adventitial lymphatics is associated with plaque progression Reduced lymphangiogenesis is associated with plaque regression Impaired lymph drainage or lymphostasis induced by lymphatic ligation promotes atherosclerotic development	[58]
Human tissue *ApoE*^*−/−*^ mice	Atherosclerotic tissue (aortic and coronary artery) Blockade of LGR4-mediated signaling LGR4 gene silencing LYVE-1, RSPO2, VEGF-C, eNOS, AKT, VEGFR-3, Ki67,	RSPO2 levels are elevated in human and mouse atherosclerotic arteries RSPO2 inhibits lymphangiogenesis via LGR4-mediated signaling and hinders VEGF-C-stimulated AKT and eNOS activation in LECs Blockade of RSPO2-LGR4 signaling attenuates atherosclerosis Inhibition of LGR4-mediated signaling increases LVD and arterial efflux of cholesterol	[59]

*α-SMA* alpha-smooth muscle actin, *eNOS* endothelial nitric oxide synthase, *FOXC-2* Forkhead Box C2, *i.p.* intraperitoneal injection, *i.v.* intravenous injection, *oxLDL* oxidized low-density lipoprotein, *SR-BI* scavenger receptor class B type I

Although the relationship between aortic atherosclerosis and hypertension has not been determined in the general population, the high blood pressure is assumed to be associated with complex atherosclerosis [60]. Recent research has implicated the role of lymphatics in the regulation of blood pressure and pathogenesis of hypertension. In salt-sensitive hypertension, a high-salt diet (HSD) in rats leads to interstitial hypertonic sodium accumulation with subsequent dermal lymphangiogenesis, suggesting that the tonicity-responsive enhancer binding protein (TonEBP)-VEGF-C signaling in macrophages and dendritic cells is a major determinant of extracellular volume and blood pressure homeostasis [61]. The mouse model of salt-sensitive hypertension has shown that renal lymphatics play a key role in immune cell trafficking and blood pressure regulation [62]. Enhanced lymphatics may aid in the clearance of interstitial immune cells, as evidenced by reduced accumulation of renal CD11c^+^F4/80^−^ monocytes. Increased renal LVD is supposed to prevent angiotensin II-induced hypertension [63]. VEGF-C administration in salt-treated mice has enhanced renal and skin lymphangiogenesis, and attenuated renal injury [64]. Recently, VEGF-Cc156s has been shown to prevent angiotensin II-induced cardiac dysfunction by improving cardiac lymphatic function, alleviating inflammation and fibrosis, and ameliorating arterial hypertension in mouse models [65]. The immune cell accumulation and interstitial fluid retention are considered to be risk factors for inducing hypertension in this field [66]. Thus, lymphangiogenesis is also an essential factor for improving blood pressure and reducing atherosclerosis risk.

Furthermore, sex differences in the physiology and pathophysiology, and even the signaling pathways of endothelial cells may have a potential impact on cardiovascular diseases, e.g., atherosclerosis and hypertension [67, 68]. In spite of their importance, however, there are very few data concerning the sex difference in LEC function and lymphangiogenesis. Previous study has shown that cardiomyocyte-specific estrogen receptor alpha (ERα) increases lymphangiogenesis and reduces fibrosis in the female mouse heart after MI [69], indicating that involvement of ERα in the enhancement of lymphangiogenesis may protect female cardiomyocytes from the sequelae of ischemia and contribute to the attenuation of cardiac remodeling. During hypertension, sex differences have been noted in renal immune cell infiltration and regulation of lymphatic responses. An increased pressor response to angiotensin II in males is associated with greater T-cell infiltration in the kidney [70]. Recent study has demonstrated that female mice with angiotensin II-induced hypertension show increased renal LVD, exhibiting differences in immune activation, inflammatory, and hormonal milieu. Females have attenuated renal T-cell accumulation and have been partially protected from the prohypertensive effects of T cells. VEGF-C expression in the female kidney has been increased in response to angiotensin II infusion [63]. In the case, genetic intervention of renal LVD may help to prevent the development of angiotensin II-induced hypertension. Moreover, sex-dependent differences have been identified in lymphatic numbers regardless of genotype, featured with more cardiac lymphatics in female mice. The female mice overexpressing adrenomedullin (*Adm*^*hi/hi*^) have significantly more cardiac lymphatics than their wild type counterparts [71]. These findings have also suggested that innate differences between the sexes may impact the lymphangiogenic repair process for preserving cardiac function and reducing edema after heart injury.

#### MI

MI, a life-threatening condition, occurs due to occlusion of the coronary artery, leading to transmural myocardial ischemia, injury, and even necrosis. The ischemia induces profound metabolic and ionic perturbations in the affected myocardium and causes rapid depression of systolic function, which further contributes to a wavefront phenomenon of cardiomyocyte death from subendocardium to subepicardium [72]. Increased microvascular permeability resulting from myocardial injury is an early event in the deterioration of myocardial vascular integrity. As a result, excessive fluid accumulates in the interstitial space of the heart, leading to acute myocardial edema [73]. Moreover, MI triggers a robust inflammatory response featured by the coordinated mobilization of different mononuclear phagocytes, which aid in scavenging dead cardiomyocytes and degrading released macromolecules for promoting granulation tissue formation and remodeling [56, 74].

VEGF-C/VEGFR-3 signaling as a key regulator of LEC proliferation, migration, and survival is involved in cardiac lymphangiogenesis and healing process. In the acute phase of MI, lymphatic dilation occurs, correlating with edema and immune cell infiltration [23]. VEGF-C secreted by the proinflammatory macrophages drives lymphangiogenesis and extensive remodeling of the cardiac lymphatic network to maintain immune cell homeostasis and effective tissue repair during the post-MI healing process in mice [7, 25]. Increased LVD has modulating effects on immune response and activity of the inflammatory process [75]. Lymphangiogenesis in MI appears to be beneficial in resolution of inflammation and edema by increasing the clearance of fluid and immune cells as well as inflammatory mediators from the injured heart to draining LNs [76]. In a rat cardiac ischemia–reperfusion model, VEGF-C protects heart from injury by inhibiting cardiomyocyte apoptosis [77]. In a mouse model, VEGFR‐3 knockdown impairs cardiac lymphangiogenesis, leading to cardiac edema after long‐term pressure overload [78], suggesting that VEGF-C/VEGFR-3 axis protects against pressure-overload induced cardiac dysfunction through regulation of lymphangiogenesis. Also, the blockage of VEGF-C signaling by soluble VEGFR-3 (sVEGFR-3) results in impaired morphology of cardiac lymphatics, increased lymphatic leakage, and raised MI-induced mortality in mice [24].

Lymphangiogenesis may reduce pathological remodeling and enhance functional recovery of the heart. Cardiac lymphangiogenesis occurs both in the infarcted and surrounding non-infarcted regions in mammalian models and humans. Circulating immune cells including activated macrophages undertake extensive phagocytic activity in the infarcted region [79]. Several studies have suggested that enhancing lymphangiogenesis following MI significantly improves myocardial function and reduces scar formation. Interstitial fluid accumulation has an impact on the organization of extracellular matrix (ECM) components in the heart, including fibronectin that signals to LECs via β1 integrins directly associating with VEGFR-3 to amplify VEGF-C signaling [17, 32]. Connective tissue growth factor (CTGF), a matricellular protein, is dispensable during cardiac injury and fibrosis, while inhibiting CTGF in activated fibroblasts is sufficient to abrogate the fibrotic response to angiotensin II [80]. Histopathological observation of human MI tissue with D2-40 immunostaining has suggested that newly formed lymphatics may be involved mainly in the maturation of fibrosis and scar formation through the drainage of excessive proteins and fluid [81]. It should be noted however that the links between interstitial fluid accumulation and functional lymphangiogenesis is complicated, depending on the local microenvironment.

Lymphangiogenesis, an essential element for cardiac homeostasis, may play a central role in facilitating repair of cardiac tissues following MI. The induction of VEGFR-3 signaling pathway as a potential target for promoting lymphangiogenesis and modulating immune response has been shown to be efficacious after MI in experimental animal models. The critical role of lymphangiogenesis in cardiac remodeling, fibrosis, and regeneration may suggest attractive therapeutic targets for MI (Table 2).

**Table 2:** Lymphangiogenesis in myocardial infarction (MI)

Animals/human	Main targets (cytokine/chemokine/growth factor) and others	Lymphangiogenic properties and lymphatic features	References
C57BL/6 mice Vegfr3^LacZ/+^ mice *Prox-1*^ fl/+^ mice	LAD ligation VEGF-C-Cys(156)Ser, i.p VEGFR-3, *Prox-1*, LYVE-1, podoplanin, GFP	Cardiac lymphatics emerge at E12.5 from extra-cardiac regions Lymphatics of neonatal hearts form an extensive branched network VEGF-C enhances cardiac lymphangiogenic response to promote functional improvement in MI	[7]
Zebrafish lines *cxcr4a mutant zebrafish*	Cryoinjury or resection (ventricle apex) *fli1a:GFP*, *flt4:mCitrine*, *prox1:Gal4-UAS:RFP*, *lyve1:RFP*	The presence of necrotic tissue and scar formation promotes lymphangiogenesis after cardiac injury Cardiac lymphatics functionally support the heart during regeneration and homeostasis Cardiac lymphangiogenesis requires VEGF-C signaling and helps reduce scar volume in response to cryoinjury	[8]
*Prox-1*-deficient mice *Reln*-deficient mice *Reln*^+*/−*^ mice *Integrin β1*^*f/f*^ mice	LAD ligation LYVE-1, Reelin, Prox1, α-SMA, Ki67	LEC-derived Reelin promotes cardiomyocyte proliferation and survival through integrin-β1 signaling Embryos that lack Reelin-producing LECs develop smaller hearts as a consequence of reduced cardiomyocyte proliferation and increased cardiomyocyte apoptosis Cardiomyocyte culture with LEC-conditioned medium indicates that LECs produce lymphoangiocrine signals for controlling cardiomyocyte homeostasis Reelin re-expression in lymphatics of the injured neonatal heart improves cardiac regeneration and function in MI Reelin improves cardiac function and reduces scar formation after adult MI	[12]
MHC class II-deficient mice Wistar rats	rhVEGF-C_C156S,_ sVEGFR-3-IgG construct, i.p LYVE-1, F4-80, CD11b, CD68, VE-cadherin	Sustained VEGF-C delivery is required for therapeutic lymphangiogenesis Lymphangiogenesis limits cardiac inflammation and dysfunction sVEGFR-3 limits T-cell levels and reduces deleterious cardiac remodeling by inhibiting lymphangiogenesis CD4^+^ and CD8^+^ T cells suppress cardiac lymphangiogenesis in MI	[13]
Apelin-KO mice C57BL/6 J mice	LAD ligation, lymphography LYVE-1, podoplanin, VE-cadherin	Chronic myocardial ischemia induces pathological lymphatic remodeling and dysfunction Apelin deficiency promotes VEGF-C expression and lymphangiogenesis in MI Lack of apelin exacerbates a proinflammatory response and worsens lymphatic abnormality in MI	[23]
sVEGFR-3 mice Chy mice	LAD ligation LYVE-1, *Prox-1*, F4/80, CD45, α-SMA	sVEGFR-3 mice display intramyocardial hemorrhages in the infarcted areas and higher mortality in MI VEGFR-3 downregulation alters cardiac lymphatic morphology with a reduced capability to respond to lymphangiogenic signals	[24]
Wistar rats	LAD ligation or occlusion, lymphangiography VEGF-C_C152S_, i.m LYVE-1, podoplanin, *Prox-1*, VEGFR-3, CCL21	Cardiac lymphatic remodeling decreases lymph transport in MI Targeted delivery of VEGF-C_C152S_ stimulates cardiac lymphangiogenesis, improves myocardial fluid balance, and attenuates cardiac inflammation, fibrosis (interstitial collagen density) and dysfunction	[25]
*Adm*^*hi/hi*^ mice *Cx43*^*fl/fl*^ mice *Vegfr3-CreER*^*T2*^ mice	LAD ligation, microlymphography LYVE-1	AM drives cardiac lymphangiogenesis in MI AM overexpression reduces edema and improves cardiac function in MI Connexin 43 deletion results in defective permeability and function of lymphatics	[71]
*Lyve1*^*–/–*^, *hCD68-EGFP* mice *R26R*^*tdTomato*^ mice *Myh6-Cre/Esr1* mice	LAD ligation or occlusion, Evans Blue dye LYVE-1, CD68, CD45, CD11b, F4/80, CD11c, CCL21, *Prox-1*, VEGFR-3	VEGF-C-driven cardiac lymphangiogenesis increases clearance of immune cells LYVE-1 is required for immune cell clearance to mediastinal LNs Disruption of LYVE-1-dependent clearance of immune cells via lymphatics is detrimental to cardiac function in MI	[79]
C57BL/6 J mice	LAD ligation or occlusion, lymphatic flow VEGF‐C_Cys156Ser_, VEGFR-3 inhibitor (MAZ‐51), VEGF-C NAb, i.m LYVE-1, TNF‐α, IL‐1β, IL‐6	Myocardial IRI stimulates endogenous lymphangiogenesis Enhancing endogenous lymphangiogenesis attenuates ischemic-induced heart failure MAZ‐51 and VEGF‐C NAb impair endogenous lymphangiogenesis and exacerbate heart failure	[82]
Human tissue *ApoE*^*−/−*^ mice *Sema3e*^*−/−*^ and *Plxnd1*^*−/−*^ mice	Whole-mount confocal images Wound healing assay *Prox-1*, VEGFR-3, LYVE-1, VEGF-C, Sema3E, PlexinD1	Sema3E-PlexinD1 contributes to cardiac lymphatic development, and induces repulsion and cytoskeletal collapse of LECs Disruption of Sema3E-PlexinD1 leads to cardiac lymphatic malformation Inhibition of Sema3E-PlexinD1 stimulates lymphangiogenesis and improves cardiac function after MI	[83]

*GFP* green fluorescent protein, *i.m.* intramyocardial, *i.p.* intraperitoneal injection, *LAD* left descending artery, *Nab* neutralizing antibody, *rhVEGF-CC156S* recombinant human VEGF-CC156S protein, *sVEGFR-3-IgG* soluble VEGFR-3-immunoglobulin

## Lymphangiogenesis and immune regulation in cardiovascular diseases

Recent studies have shown that lymphangiogenesis and immune cell trafficking play a crucial role in some cardiovascular diseases, e.g., atherosclerosis and MI. The interplay between cardiac lymphangiogenesis and immune response to ischemic injury is a complicated sequential process. Lymphatic function and remodeling could be impacted by proinflammatory mediators [84, 85], or by different cell populations characterized by infiltrating immune cells like macrophages, DCs, B and T cells [51, 86, 87]. The potential understanding of immune cells in the regulation of lymphangiogenesis and lymphatic function will help to alleviate myocardial edema and inflammation process.

In the cardiac ischemic injury, recruited inflammatory cells including neutrophils and monocytes/macrophages phagocytose dead cells and tissue debris, in which inflammatory mediators are released to stimulate the regenerative process and induce cardiac remodeling and fibrosis, leading to a dramatic degradation of function [88, 89]. Although MI has induced robust intramyocardial lymphangiogenesis in rat models by occlusion/ligation of the coronary artery, adverse remodeling and dysfunction of collecting lymphatics result in expansion of the subepicardial lymphatic network and reduced lymph transport capacity [25]. Myocardial edema and inadequate immune response triggered by certain factors released by immune cells including macrophages may have increased collagen synthesis by fibroblasts leading to fibrosis development [90]. Genetic deletion of transient receptor potential vanilloid 4 (TRPV4) channels attenuates TGF‑β1‑induced differentiation in cardiac fibroblasts and protects heart from MI-induced adverse remodeling [91]. In adult rodents, endogenous lymphangiogenesis in response to cardiac injury seems to be insufficient to clear interstitial fluid and immune cell accumulation, and thus a drug-mediated manipulation of the lymphatic response could help to modulate the inflammatory content in the myocardium and promote both myocardial survival and restoration [11].

Following MI, the innate immune system directs the phagocytosis of cellular debris for stimulating cell repopulation and tissue renewal. The persistent influx of immune cells, coupled with the lack of an inherent regenerative capacity, may cause cardiac fibrosis in the mammalian adult heart. In Lyve1^−/−^ mutant mice, impaired immune cell uptake and clearance have resulted in a progressive deterioration in heart function, accompanied by elevated pathological remodeling and fibrosis [79]. The immune cells contribute to lymphatic remodeling and dysfunction by stimulating or inhibiting lymphangiogenesis linking to cardiovascular diseases. MI causes a unique reaction of the innate immune system in which neutrophils influx into the injury site attracted by apoptotic signals released by dying cells, especially, neutrophil recruitment into reactive LNs plays a role in programming adaptive immunity [16]. Neutrophils expressing programmed death-ligand-1 (PD-L1), arginase-1 (ARG-1) and CD10 have been reported to possess potent immunosuppressive activity [92]. Macrophages as an important component of the innate immune system, are not only involved in tissue inflammation and repair, but also support organogenesis during heart development. Cardiac lymphatics respond to MI by re-activating gene expression program for lymphangiogenesis, stimulation of this process enhances resolution of macrophage-driven inflammation and promotes tissue repair [79]. M1 macrophages characterized by the inflammatory phenotype produce cytokines such as interleukin (IL)-1β, IL-6, and tumor necrosis factor (TNF)-α, while alternatively activated M2 macrophages are anti-inflammatory and may activate fibroblasts, induce cell proliferation, collagen deposition, and angiogenesis [93–95]. In peripheral tissues, antigen‐bearing DCs and macrophages enter local lymphatics via endothelial junctions and continue to travel to regional LNs [96]. Tissue-resident macrophages are involved in regulating the growth, patterning, and expansion during cardiac lymphatic formation. The distribution and prevalence of resident macrophages in the subepicardial compartment of the developing heart coincides with the emergence of lymphangiogenesis, closely interacting with the nascent initial lymphatics [29]. Moreover, MI is associated with the increase of conventional DCs and monocyte-derived CD64^+^ CD11c^+^ major histocompatibility complex (MHC) II^+^ DCs in the heart. DCs in the ischemic heart are activated and loaded with self-antigen, licensing DCs for efficient autoreactive T cell activation, suggesting that prevention of DC maturation and self-antigen presentation could limit the onset of pathological cardiac autoimmunity following mouse MI [97]. During MI, proinflammatory milieu creates conditions favorable for activation of autoreactive clones of T cells, which may trigger autoimmune destruction of the myocardial tissue [98]. T cells may participate in microvascular obstruction during ischemia–reperfusion injury (IRI), release inflammatory cytokines and increase cellular infiltration, even further leading to the aggravation of myocardial injury [99]. The presence of CD4^+^ T cells that can be activated by autoantigens presented by MHC class II molecules is a prerequisite for proper cardiac healing, because absence of CD4^+^ T cells is associated with poorer outcome in CD4 deficient and MHC class II-deficient mice [95, 100].

Atherosclerosis is driven by the accumulation of immune cells and cholesterol in the arterial wall. Within the plaque, many processes occur for striking a delicate balance between immune and inflammatory responses, especially through the activation of Toll-like receptor 7 (TLR7). TLR7 stimulation can ameliorate atherosclerotic lesion burden and reduce plasma cholesterol in Apoe^−/−^ mice, associating with an atheroprotective B cell and Treg response [101]. Both B and T cell populations are important players with regard to the adaptive immune response in atherosclerosis. Lymphatic dysfunction appears to have a systemic effect on B cell homing and to impair lipoprotein metabolism for promoting atherosclerosis [102, 103]. Recent studies using Apoe^−/−^ mouse models have shown that resident cell interaction with LECs and innate lymphoid cells, especially immune cells including macrophages, T and B cells appears crucial at the early stage of atherosclerosis [104]. The follicular regulatory helper T cells, as a key regulator of atherosclerosis, induce expansion of anti-atherogenic regulatory B cell populations, and regulate lymphangiogenesis through IL-4 secretion in advanced atherosclerosis [103]. LECs are essential for regulating the trafficking of DCs and macrophages, and activated T cell migratory egress. LECs of the LNs can efficiently scavenge and present peripheral antigens on MHCI to induce tolerance by specific deletion of autoreactive CD8^+^ T cells, and phagocytose and process exogenous antigen and cross-present it to naïve CD8^+^ T cells. Moreover, DCs migrating to the draining LNs within Clever-1-positive lymphatics experience immunosuppressive interaction with LECs [105]. Early atherosclerotic plaque formation is associated to collecting lymphatic dysfunction to propel lymph forward in a non-specific cholesterol- but LDL-cholesterol receptor (LDLR)-dependent manner [106]. Besides, arterial lymphatics are necessary to remove cholesterol via high-density lipoprotein (HDL)-mediated RCT and egress of inflammatory cells from the atherosclerotic wall [43, 58]. Therefore, it still remains unknown as to how lymphangiogenesis plays a role in the pathophysiological progression of atherosclerosis, especially in macrophage RCT and immune cell trafficking.

## Lymphangiogenesis and heart transplantation

Fundamental research into the molecular mechanism underlying biological functions of VEGF-C and VEGF-D has been central to identifying the potential role of lymphangiogenic factors during inflammation and wound healing. Lymphangiogenesis is involved in the clearance of inflammatory cells from the injured tissues, which is a vital step in the resolution of inflammation and prevention of fibrotic remodeling [107]. However, whether lymphangiogenesis is beneficial or detrimental to graft survival is not clear [108]. Lymphangiogenesis has been proposed to facilitate chronic rejection of transplanted tissue in a range of clinical settings [109]. As a risk factor for graft rejection, it contributes to induction of long-term tolerance to the allograft and chronic alloimmune response by promoting escape of antigen-presenting cells (APCs) to regional LNs and enhancing allosensitization [110]. Increased lymphatics may enhance antigen presentation and subsequent adaptive immune response for inducing organ rejection, but decreased lymphatics may result in edema followed by acute organ rejection in many cases [111].

During organ transplantation, fine lymphatics are not adapted for surgical anastomosis, lymph drainage has been suggested to be dependent on newly formed or at least newly connected structures [112]. An immune response is triggered when the trafficking of APCs has been facilitated via functional lymphatics to the draining LNs, whereas lymphangiogenesis seems to be involved in maintenance of a detrimental alloreactive immune response. The priming of the alloimmune response may coincide with the transplant period when lymphatics are anastomosed between donor and recipient [113]. In addition, cardiac lymphatic remodeling may be an important factor affecting cardiac allograft vasculopathy (CAV) and rejection responses besides acute allograft dysfunction [114]. In human kidney transplants, lymphangiogenesis has been shown to be associated with immunologically active lymphocytic infiltrates. The nodular infiltrates have the potential to launch and perpetuate specific immune responses to graft alloantigens and thus could contribute to recurrent episodes of acute rejection [115]. In human cardiac allograft biopsy, the subendocardial inflammatory infiltrate is of recipient origin showing significant lymphangiogenesis [112]. In the context, serum level of VEGF-C may associate with risk of adverse events for providing useful information after heart transplantation [116].

Chronic rejection in allograft recipients is associated with cardiac lymphangiogenesis, which is probably an important factor in promoting cellular trafficking, alloimmunity, and CAV. Interplay of innate and adaptive immunity after heart transplantation, may result in alloimmune response detrimental to cardiac allografts and transplant recipients. Inhibition of lymphangiogenesis treated with adenoviral VEGFR-3-Ig protects allogeneic cardiac allografts against rejection by limiting lymphatic activation and transport of activated APCs [20]. VEGF-C/VEGFR-3 signaling may participate in immune cell traffic from peripheral tissues to secondary lymphoid organs by reducing CCL21 production and CD8^+^ effector cell entry in the allograft [20], suggesting VEGFR-3 inhibition could be used as a lymphatic targeted immunomodulatory therapy to regulate alloimmune activation and to improve cardiac survival. During cardiac allograft IRI in rats, stimulation of VEGF-C/VEGFR-3 axis results in early lymphatic activation and later increases in allograft inflammation. However, inhibition of the signaling pathway decreases early lymphatic activation with subsequent attenuation of acute and chronic inflammatory response, and rejection of the allograft through affecting both innate and adaptive immune responses [19]. Moreover, in rat tracheal transplantation, inhibition of VEGF-C activity with AdVEGFR-3-Ig has been shown to limit adaptive immune responses and reduce graft rejection [117]. In a mouse corneal allotransplantation model, soluble VEGFR-3 suppresses both lymphangiogenesis and immune rejection to enhance allograft survival [118]. In the model of islet allotransplantation, lymphangiogenesis occurs in islet allografts and draining LNs, and interference of lymphatic function using diverse inhibitors FTY720, sunitinib and anti-VEGFR-3 mAbs has led to inhibition of lymphangiogenesis and prolonged allograft survival [119].

Allografts introduce genetically different tissues to the recipient, which typically cause T cell mediated immune response leading to rejection and destruction of the transplant [120]. The interaction between LECs and APCs can be altered by different inflammatory and lymphangiogenic factors, which may actively influence the function of immune cells [121]. LECs can suppress T cell responses directly by presenting antigen in tolerogenic fashion or indirectly by inhibiting the maturation of DCs [122]. VEGF-trap has significantly inhibited immune cell infiltration including macrophages and CD3^+^ T cells, and improved long-term graft survival in high-risk corneal transplantation. Also, anti-VEGF-C and sVEGFR-3 significantly decreased graft lymphangiogenesis and lymphoid Th1 cell frequencies [123]. Following transplantation of a minor antigen (HY) gender-mismatched mouse cardiac graft, the enhanced lymphatic flow index (LFI) was found to be correlated with an increase in LVD, lymphatic area, and inflammatory infiltration of CD4^+^, CD8^+^ T cells, and CD68^+^ macrophages [108]. In the heterotopically transplanted rat heart, chronic rejection increases VEGF-C^+^ inflammatory cells and LYVE-1^+^ LVD, whereas chronic allograft inflammation is a potent stimulus for myocardial lymphangiogenesis via highly expressed VEGF-C mainly derived from macrophages and CD4^+^ T cells [20]. In a mouse heart transplantation model, the targeted delivery of microparticles containing tacrolimus, a T cell-specific calcineurin inhibitor, to the draining LNs of recipients has greatly prolonged allograft survival [124]. Therefore, increased evidence may provide a lymphatic delivery platform for understanding the crosstalk between cellular stress and inflammation, and between innate and adaptive immunity. The evaluation of the occurrence and time course of lymphangiogenesis after organ transplantation will help to improve graft survival in animal models [110].

Lymphangiogenesis in allografts however is not necessarily harmful and may in fact promote the resolution of inflammation. Unlike the case for hearts, islets, and corneas, lymphatic migration of APCs from pulmonary grafts to the periphery is not critical for graft rejection [18, 125, 126]. Lymphatic drainage from bronchus-associated lymphoid tissue in tolerant lung allografts promotes peripheral tolerance, and the survival of heart allografts is just dependent on the drainage from the lung allograft to the periphery [126]. Induction of lymphangiogenesis with VEGF-C_156S_ has been shown to mitigate acute rejection of mouse lung allografts, which is supposed to be associated with augmented hyaluronan clearance [127]. Clearly, the role of lymphangiogenesis on allograft rejection addressed here is deeply challenging, especially the contribution of LECs and draining LNs to transplant immunology remains controversial and limited. Table 3 has summarized the properties of LECs from available publications on heart transplantation. It can be proposed that lymphangiogenesis may constitute an important bridge for understanding the interrelation between lymphatic biology and transplant outcomes (Table 3).

**Table 3:** Lymphangiogenesis in heart transplantation

Animals/human	Main targets (cytokine/chemokine/growth factor) and others	Lymphangiogenic properties and lymphatic features	References
DA rats (syngeneic allografts) *Vegfr3*^*iΔLEC*^ mice (donor)	VEGF-C 156S, VEGF-C/D trap, VEGFR-3 NAb, i.p LYVE-1, *Prox-1*, OX62, ICAM-1, IFN-γ, TLR, TNF-α	Ischemia increases VEGF-C mRNA levels and proportion of VEGFR-3^+^/OX62^+^ donor DCs IRI-related innate immune response is required for LEC activation VEGF-C enhances LEC activation and chemokine expression Allografts treated with VEGF-C/D trap exhibit less LEC activation and DC maturation VEGF-C/D trap reduces alloimmune response, chronic inflammation, allograft vasculopathy and cardiac fibrosis Inhibition of VEGFR-3 prolongs cardiac allograft survival	[19]
DA rats (heterotopic allografts) BALB/c donor mice C57/BL6J recipient mice	Ad.VEGFR-3-Ig (VEGF-C/D-Trap), p.v.p VEGFR-3-NAb (mF4–31C1), i.p LYVE-1, *Prox-1*, VEGF-C, VEGFR-3, CD4, CD8, CD11b, CCL-21, IFN-γ	Chronic rejection induces myocardial lymphangiogenesis VEGF-C is mainly derived from macrophages and CD4^+^ T cells, and VEGFR-3^+^ LECs are donor derived sVEGFR-3-Ig improves cardiac allograft survival VEGFR-3 inhibition decreases CD8^+^ effector cell recruitment to allograft and LEC-derived CCL21 production, and alloimmune response VEGFR-3-NAb decreases lymphatic activation and allograft inflammation	[20]
C57BL/6 (H-2^b^) mice (heterotopic allografts)	Lymphoscintigraphy Evan's Blue dye LYVE-1, CD4, CD8, CD68	Chronic rejection is associated with myocardial lymphangiogenesis Donor cells are the main source of lymphangiogenesis CAV correlates with increased lymphatic area and infiltrating CD8^+^ T cells Lymph flow correlates with lymphatic area, but not LVD Increased lymph flow and lymphatic area promotes inflammatory infiltrate in allografts	[108]
Human (cardiac transplant recipients)	CAV classification VEGF-A, VEGF-C, PF-4	Soluble proteins, e.g., VEGF-A/-C are involved in endothelial injury, repair and proliferation in established CAV VEGF-C, VEGF-A and PF4 are sensitive and specific biomarkers of CAV	[114]

*DA* Dark Agouti, *IFN-γ* interferon γ, *ICAM-1* intracellular adhesion molecule-1, *i.p.* intraperitoneal injection, *PF-4* platelet factor-4, *p.v.p.* portal venous perfusion

## Prospective therapeutic strategies targeting lymphangiogenesis in cardiovascular diseases

Modulation of lymphangiogenesis has recently emerged as a new platform for investigating therapeutic strategies in cardiovascular diseases. Lymph transport capacity in MI and atherosclerosis has not been sufficient to control excessive leakage of fluid and proteins to the interstitial tissue due to vascular hyperpermeability. Myocardial injury initiates a robust immune response, characterized by sequential mobilization of monocytes in cardiac healing [74]. In particular, lymphangiogenesis in inflammatory settings promotes macrophage mobilization and facilitates the resolution of insufficient cardiac lymph drainage [3, 87]. Impaired cardiac lymphatics however lead to myocardial edema and delayed immune responses, which in turn may have further negative impact on cardiac structure and function.

Recent studies have shown that selective regulation of cardiac lymphangiogenesis should be focused on resolving edema formation, inflammatory cell accumulation and fibrosis [78, 128]. So far, VEGF-C/VEGFR-3 has been considered as the most important and promising candidate for lymphangiogenic therapy in this field, but different administration routes may account for inconsistent results in protein and gene-based therapies. To develop safe and effective approaches for stimulating cardiac lymphangiogenesis in post-MI, much effort has been on sustained protein release or gene delivery using adeno-associated virus (AAV) vectors. In mouse and rat post-MI, stimulation of lymphangiogenesis with targeted intramyocardial delivery of recombinant VEGF-C protein has accelerated resorption of chronic myocardial inflammation and edema, reduced deleterious cardiac remodeling, and improved cardiac function [7, 25, 79]. In a mouse myocardial IRI model, lymphangiogenesis stimulated with VEGF-C delivered by gelatin hydrogel placed on the epicardium has also shown reduced inflammation, edema and fibrosis, as well as increased cardiac function. Blocking VEGF-C with a neutralizing antibody has blunted the increase in LVD and lymph transport, and exacerbated cardiac injury and dysfunction [82]. In the acute and chronic phases of repair and recovery after mouse MI, therapeutic lymphangiogenesis via AAV-VEGF-C_C156S_ gene delivery has prevented cardiac lymphatic reduction, and limited cardiac inflammation and dysfunction, but conversely, AAV-sVEGFR-3 (soluble VEGFR-3, VEGF-C/VEGF-D trap) has inhibited infarct lymphangiogenesis and reduced T-cell infiltration and deleterious cardiac remodeling [13].

Other signaling pathways and factors have been reported to improve cardiac function after MI by regulating lymphangiogenesis. Apelin, a bioactive peptide promotes lymphatic development in zebrafish [6] and intratumoral lymphangiogenesis in mice [129], and potently improves cardiac contractility and reduces cardiac loading in pathological settings [130]. In an apelin-knockout mouse model, abnormally dilated and leaky lymphatics have shown to be associated with a proinflammatory status after MI, but the overexpression of apelin in ischemic heart has shown to be sufficient to restore functional lymphatics and to reduce matrix remodeling and inflammation [23]. The extracellular matrix protein reelin (RELN), a lymphoangiocrine signal produced by LECs has been shown to control proliferation and survival of cardiomyocytes during mouse development, and to protect cardiomyocytes from apoptosis, which correlates with reduced scar and improved heart function [12]. The efficient cardiac repair after MI in neonatal and adult mice has been contributed to RELN-induced cardioprotective effect and cardiac regeneration [12]. Adrenomedullin (AM), a highly potent vasodilator peptide, is essential for proper cardiovascular and lymphatic development in mice [131]. Overexpressed AM induces cardiac lymphangiogenesis after MI, resulting in improved cardiac function and reduced myocardial edema via regulation of connexin 43 [71]. In post-infarct heart failure rat models, cardiac fibroblasts expressing vascular cell adhesion molecule-1 (VCAM-1) improve heart function by triggering lymphangiogenesis [14]. A combined delivery of lymphatic endothelial progenitor cells (LEPCs) and VEGF-C with the functionalized self-assembling peptide (SAP), or delivery of SAP-thymosin β4 (Tβ4) has shown to be effective for enhancing cardiac lymphangiogenesis after MI, reducing cardiac inflammation and edema, attenuating reverse myocardial remodeling, and improving cardiac function and repair of the infarcted myocardium [132, 133]. A recent study has shown that the epicardium and pericardium-derived Semaphorin 3E (Sema3E) and its receptor, PlexinD1, are essential for the coronary stem formation and cardiac lymphatic development. Inactivation of Sema3E-PlexinD1 signaling has improved the recovery of cardiac function by increasing lymphangiogenesis in an adult mouse model of MI [83], suggesting a therapeutic possibility for targeting Sema3E-PlexinD1 signaling in cardiovascular diseases.

Furthermore, increased lymphatic function is associated with improved clearance of excess cholesterol from peripheral tissues, including the arterial wall, the major site of atherosclerotic plaque formation, and thus inhibition of lymph drainage exacerbates atherosclerosis. Lymphangiogenic therapy may accelerate RCT and alleviate inflammatory responses, leading to regression or inhibition of atherosclerosis [76]. In human and mouse atherosclerotic arteries, increased R-spondin 2 (RSPO2) inhibits lymphangiogenesis via leucine-rich repeat-containing G protein-coupled receptor 4 (LGR4) and downstream impairment of protein kinase B (PKB/AKT)-endothelial nitric oxide synthase (eNOS) signaling [59]. The regulation of the signaling pathways may provide a therapeutic option for improving cholesterol efflux from atherosclerotic arteries by stimulating lymphangiogenesis. Epsins, a family of adaptor proteins involved in clathrin-dependent endocytosis, control VEGFR-3 degradation in LECs for regulating lymphangiogenesis, lymphatic maturation and valve formation [134, 135], designing molecules to target increased expression of macrophage epsins may represent a therapeutic strategy to treat atherosclerosis [15].

Targeting the synergy between lymphangiogenesis and inflammatory infiltration is beneficial for promoting immune modulation and cardiac repair. Although gene therapy holds a great promise for future treatment of cardiovascular diseases, the way either by intramyocardial injection or intravascular infusion to deliver the therapy remains a challenge. As a matter of delivery, viral vectors vary greatly in range of infectivity, transgene capacity and expression stability. Although tissue specific AAVs can increase therapeutic transgene levels even up to one year, adenoviruses and plasmids provide only short-term transgene expression. Also, neutralizing antibodies might limit the therapeutic effect and cause a detrimental immune response [136]. Therefore, behind the gene therapeutic processes, great attention should be paid on the basic biology and fundamental aspects.

## Conclusions and perspectives

**Fig. 3:**
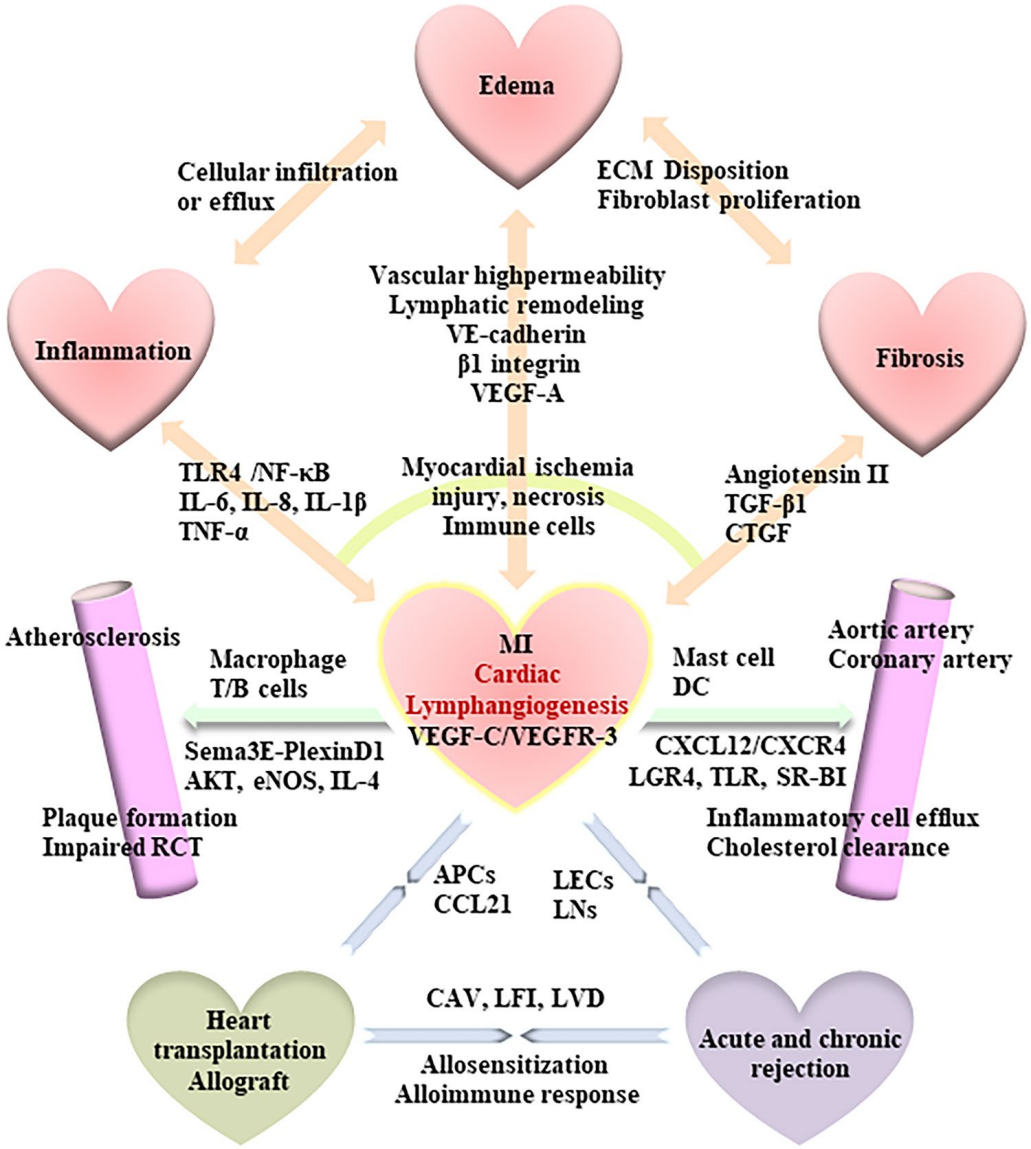
Schematic diagram depicting the lymphangiogenesis regulated by several kinds of cytokines, chemokines, and growth factors has emerged as a major therapeutic or interventional target in cardiovascular diseases and heart transplantation

Cardiac lymphangiogenesis plays an important role in improved resolution of inflammation, edema and fibrosis, and ameliorated cardiac dysfunction, which has emerged as a major therapeutic goal in cardiovascular diseases (Fig. 3). However, regarding the positive and negative impacts of lymphangiogenesis in the pathological processes of MI and atherosclerosis, as well as heart transplant rejection, several questions need to be addressed in the future study. What are the connection points among atherosclerosis, MI and heart transplantation in the field of lymphangiogenesis? How activated immune cells in corporation with lymphangiogenic mediators contribute to the pathological process of common cardiovascular diseases? How do the chemokines, cytokines, and growth factors bridge lymphangiogenesis and innate/adaptive immunity during heart transplantation or allograft IRI? Obviously, further understanding of the molecular mechanism of cardiac lymphangiogenesis may offer new possibilities for therapeutic interventions in cardiovascular diseases and heart transplantation.

## References

[1] Ji RC, Kato S (2001). Histochemical analysis of lymphatic endothelial cells in lymphostasis. Microsc Res Tech.

[2] Guo R, Zhou Q, Proulx ST, Wood R, Ji RC, Ritchlin CT, Pytowski B, Zhu Z, Wang YJ, Schwarz EM, Xing L (2009). Inhibition of lymphangiogenesis and lymphatic drainage via vascular endothelial growth factor receptor 3 blockade increases the severity of inflammation in a mouse model of chronic inflammatory arthritis. Arthritis Rheum.

[3] Huggenberger R, Siddiqui SS, Brander D, Ullmann S, Zimmermann K, Antsiferova M, Werner S, Alitalo K, Detmar M (2011). An important role of lymphatic vessel activation in limiting acute inflammation. Blood.

[4] Ji RC, Eshita Y, Kobayashi T, Hidano S, Kamiyama N, Onishi Y (2018). Role of simvastatin in tumor lymphangiogenesis and lymph node metastasis. Clin Exp Metastasis.

[5] Jackson DG (2019) Leucocyte Trafficking via the Lymphatic Vasculature- Mechanisms and Consequences. Front Immunol 10:471. 10.3389/fimmu.2019.00471 10.3389/fimmu.2019.00471 PMC6426755 30923528

[6] Kim JD, Kang Y, Kim J, Papangeli I, Kang H, Wu J, Park H, Nadelmann E, Rockson SG, Chun HJ, Jin SW (2014). Essential role of Apelin signaling during lymphatic development in zebrafish. Arterioscler Thromb Vasc Biol.

[7] Klotz L, Norman S, Vieira JM, Masters M, Rohling M, Dubé KN, Bollini S, Matsuzaki F, Carr CA, Riley PR (2015). Cardiac lymphatics are heterogeneous in origin and respond to injury. Nature.

[8] Harrison MR, Feng X, Mo G, Aguayo A, Villafuerte J, Yoshida T, Pearson CA, Schulte-Merker S, Lien CL (2019) Late developing cardiac lymphatic vasculature supports adult zebrafish heart function and regeneration. Elife 8 10.7554/eLife.42762 PMC6881116 31702553

[9] Aurora AB, Porrello ER, Tan W, Mahmoud AI, Hill JA, Bassel-Duby R, Sadek HA, Olson EN (2014). Macrophages are required for neonatal heart regeneration. J Clin Invest.

[10] Merz SF, Korste S, Bornemann L, Michel L, Stock P, Squire A, Soun C, Engel DR, Detzer J, Lörchner H, Hermann DM, Kamler M, Klode J, Hendgen-Cotta UB, Rassaf T, Gunzer M, Totzeck M (2019) Contemporaneous 3D characterization of acute and chronic myocardial I/R injury and response. Nat Commun 10:2312. 10.1038/s41467-019-10338-2 10.1038/s41467-019-10338-2 PMC6534576 31127113

[11] Klaourakis K, Vieira JM, Riley PR (2021) The evolving cardiac lymphatic vasculature in development, repair and regeneration. Nat Rev Cardiol 18:368–379. 10.1038/s41569-020-00489-x 10.1038/s41569-020-00489-x PMC7812989 33462421

[12] Liu X, De la Cruz E, Gu X, Balint L, Oxendine-Burns M, Terrones T, Ma W, Kuo HH, Lantz C, Bansal T, Thorp E, Burridge P, Jakus Z, Herz J, Cleaver O, Torres M, Oliver G (2020) Lymphoangiocrine signals promote cardiac growth and repair. Nature 588:705–711. 10.1038/s41586-020-2998-x 10.1038/s41586-020-2998-x PMC7770123 33299187

[13] Houssari M, Dumesnil A, Tardif V, Kivelä R, Pizzinat N, Boukhalfa I, Godefroy D, Schapman D, Hemanthakumar KA, Bizou M, Henry JP, Renet S, Riou G, Rondeaux J, Anouar Y, Adriouch S, Fraineau S, Alitalo K, Richard V, Mulder P, Brakenhielm E (2020) Lymphatic and immune cell cross-talk regulates cardiac recovery after experimental myocardial infarction. Arterioscler Thromb Vasc Biol 40:1722–1737. 10.1161/ATVBAHA.120.314370 10.1161/ATVBAHA.120.314370 PMC7310303 32404007

[14] Iwamiya T, Segard BD, Matsuoka Y, Imamura T (2020) Human cardiac fibroblasts expressing VCAM1 improve heart function in postinfarct heart failure rat models by stimulating lymphangiogenesis. PLoS One 15:e0237810. 10.1371/journal.pone.0237810 10.1371/journal.pone.0237810 PMC7494079 32936824

[15] Bhattacharjee S, Lee Y, Zhu B, Wu H, Chen Y, Chen H (2021) Epsins in vascular development, function and disease. Cell Mol Life Sci 78:833–842. 10.1007/s00018-020-03642-4 10.1007/s00018-020-03642-4 PMC7902377 32930806

[16] Oliver G, Kipnis J, Randolph GJ, Harvey NL (2020) The lymphatic vasculature in the 21st century: novel functional roles in homeostasis and disease. Cell 182:270–296. 10.1016/j.cell.2020.06.039 10.1016/j.cell.2020.06.039 PMC7392116 32707093

[17] Brakenhielm E, González A, Díez J (2020) Role of cardiac lymphatics in myocardial edema and fibrosis: JACC review topic of the week. J Am Coll Cardiol 76:735–744. 10.1016/j.jacc.2020.05.076 10.1016/j.jacc.2020.05.076 32762908

[18] Lakkis FG, Arakelov A, Konieczny BT, Inoue Y (2000) Immunologic 'ignorance' of vascularized organ transplants in the absence of secondary lymphoid tissue. Nat Med 6:686–688. 10.1038/76267 10.1038/76267 10835686

[19] Dashkevich A, Raissadati A, Syrjälä SO, Zarkada G, Keränen MA, Tuuminen R, Krebs R, Anisimov A, Jeltsch M, Leppänen VM, Alitalo K, Nykänen AI, Lemström KB (2016) Ischemia-reperfusion injury enhances lymphatic endothelial VEGFR3 and rejection in cardiac allografts. Am J Transplant 16:1160–1172. 10.1111/ajt.13564 10.1111/ajt.13564 26689983

[20] Nykänen AI, Sandelin H, Krebs R, Keränen MA, Tuuminen R, Kärpänen T, Wu Y, Pytowski B, Koskinen PK, Ylä-Herttuala S, Alitalo K, Lemström KB (2010) Targeting lymphatic vessel activation and CCL21 production by vascular endothelial growth factor receptor-3 inhibition has novel immunomodulatory and antiarteriosclerotic effects in cardiac allografts. Circulation 121:1413–1422. 10.1161/CIRCULATIONAHA.109.910703 10.1161/CIRCULATIONAHA.109.910703 20231530

[21] Zhu J, Inomata T, Fujimoto K, Uchida K, Fujio K, Nagino K, Miura M, Negishi N, Okumura Y, Akasaki Y, Hirosawa K, Kuwahara M, Eguchi A, Shokirova H, Yanagawa A, Midorikawa-Inomata A, Murakami A (2021) Ex vivo-induced bone marrow-derived myeloid suppressor cells prevent corneal allograft rejection in mice. Invest Ophthalmol Vis Sci 62:3. 10.1167/iovs.62.7.3 10.1167/iovs.62.7.3 PMC8185403 34061951

[22] Ratajska A, Gula G, Flaht-Zabost A, Czarnowska E, Ciszek B, Jankowska-Steifer E, Niderla-Bielinska J, Radomska-Lesniewska D (2014) Comparative and developmental anatomy of cardiac lymphatics. ScientificWorldJournal 2014:183170. 10.1155/2014/183170 10.1155/2014/183170 PMC3926219 24592145

[23] Tatin F, Renaud-Gabardos E, Godet AC, Hantelys F, Pujol F, Morfoisse F, Calise D, Viars F, Valet P, Masri B, Prats AC, Garmy-Susini B (2017) Apelin modulates pathological remodeling of lymphatic endothelium after myocardial infarction. JCI Insight 2:e93887. 10.1172/jci.insight.93887 10.1172/jci.insight.93887 PMC5470877 28614788

[24] Vuorio T, Ylä-Herttuala E, Laakkonen JP, Laidinen S, Liimatainen T, Ylä-Herttuala S (2018) Downregulation of VEGFR3 signaling alters cardiac lymphatic vessel organization and leads to a higher mortality after acute myocardial infarction. Sci Rep 8:16709. 10.1038/s41598-018-34770-4 10.1038/s41598-018-34770-4 PMC6232169 30420641

[25] Henri O, Pouehe C, Houssari M, Galas L, Nicol L, Edwards-Lévy F, Henry JP, Dumesnil A, Boukhalfa I, Banquet S, Schapman D, Thuillez C, Richard V, Mulder P, Brakenhielm E (2016) Selective stimulation of cardiac lymphangiogenesis reduces myocardial edema and fibrosis leading to improved cardiac function following myocardial infarction. Circulation 133:1484–1497. 10.1161/CIRCULATIONAHA.115.020143 10.1161/CIRCULATIONAHA.115.020143 26933083

[26] Ji RC (2018) Recent advances and new insights into muscular lymphangiogenesis in health and disease. Life Sci 211:261–269. 10.1016/j.lfs.2018.09.043 10.1016/j.lfs.2018.09.043 30261160

[27] Flaht-Zabost A, Gula G, Ciszek B, Czarnowska E, Jankowska-Steifer E, Madej M, Niderla-Bielińska J, Radomska-Leśniewska D, Ratajska A (2014) Cardiac mouse lymphatics: developmental and anatomical update. Anat Rec (Hoboken) 297:1115–1130. 10.1002/ar.22912 10.1002/ar.22912 24700724

[28] Lioux G, Liu X, Temiño S, Oxendine M, Ayala E, Ortega S, Kelly RG, Oliver G, Torres M (2020) A second heart field-derived vasculogenic niche contributes to cardiac lymphatics. Dev Cell 52:350–363.e6. 10.1016/j.devcel.2019.12.006 10.1016/j.devcel.2019.12.006 PMC7374559 31928974

[29] Cahill TJ, Sun X, Ravaud C, Villa Del Campo C, Klaourakis K, Lupu IE, Lord AM, Browne C, Jacobsen SEW, Greaves DR, Jackson DG, Cowley SA, James W, Choudhury RP, Vieira JM, Riley PR (2021) Tissue-resident macrophages regulate lymphatic vessel growth and patterning in the developing heart. Development 148:dev194563. 10.1242/dev.194563 10.1242/dev.194563 PMC7875498 33462113

[30] Kholová I, Dragneva G, Cermáková P, Laidinen S, Kaskenpää N, Hazes T, Cermáková E, Steiner I, Ylä-Herttuala S (2011) Lymphatic vasculature is increased in heart valves, ischaemic and inflamed hearts and in cholesterol-rich and calcified atherosclerotic lesions. Eur J Clin Invest 41:487–497. 10.1111/j.1365-2362.2010.02431.x 10.1111/j.1365-2362.2010.02431.x 21128936

[31] Emini Veseli B, Perrotta P, De Meyer GRA, Roth L, Van der Donckt C, Martinet W, De Meyer GRY (2017) Animal models of atherosclerosis. Eur J Pharmacol 816:3–813. 10.1016/j.ejphar.2017.05.010 10.1016/j.ejphar.2017.05.010 28483459

[32] Urner S, Planas-Paz L, Hilger LS, Henning C, Branopolski A, Kelly-Goss M, Stanczuk L, Pitter B, Montanez E, Peirce SM, Mäkinen T, Lammert E (2019) Identification of ILK as a critical regulator of VEGFR3 signalling and lymphatic vascular growth. EMBO J 38:e99322. 10.15252/embj.201899322 10.15252/embj.201899322 PMC6331728 30518533

[33] Cimini M, Cannatá A, Pasquinelli G, Rota M, Goichberg P (2017) Phenotypically heterogeneous podoplanin-expressing cell populations are associated with the lymphatic vessel growth and fibrogenic responses in the acutely and chronically infarcted myocardium. PLoS One 12:e0173927. 10.1371/journal.pone.0173927 10.1371/journal.pone.0173927 PMC5363820 28333941

[34] Galkina E, Ley K (2009) Immune and inflammatory mechanisms of atherosclerosis (*). Annu Rev Immunol 27:165–197. 10.1146/annurev.immunol.021908.132620 10.1146/annurev.immunol.021908.132620 PMC2734407 19302038

[35] Rafieian-Kopaei M, Setorki M, Doudi M, Baradaran A, Nasri H (2014). Atherosclerosis: process, indicators, risk factors and new hopes. Int J Prev Med.

[36] Randolph GJ, Miller NE (2014) Lymphatic transport of high-density lipoproteins and chylomicrons. J Clin Invest 124:929–935. 10.1172/JCI71610 10.1172/JCI71610 PMC3934183 24590278

[37] Ouimet M, Barrett TJ, Fisher EA (2019) HDL and reverse cholesterol transport. Circ Res 124:1505–1518. 10.1161/CIRCRESAHA.119.312617 10.1161/CIRCRESAHA.119.312617 PMC6813799 31071007

[38] Huang LH, Elvington A, Randolph GJ (2015) The role of the lymphatic system in cholesterol transport. Front Pharmacol 6:182. 10.3389/fphar.2015.00182 10.3389/fphar.2015.00182 PMC4557107 26388772

[39] Drozdz K, Janczak D, Dziegiel P, Podhorska M, Patrzałek D, Ziółkowski P, Andrzejak R, Szuba A (2008) Adventitial lymphatics of internal carotid artery in healthy and atherosclerotic vessels. Folia Histochem Cytobiol 46:433–436. 10.2478/v10042-008-0083-7 10.2478/v10042-008-0083-7 19141394

[40] Rademakers T, van der Vorst EP, Daissormont IT, Otten JJ, Theodorou K, Theelen TL, Gijbels M, Anisimov A, Nurmi H, Lindeman JH, Schober A, Heeneman S, Alitalo K, Biessen EA (2017) Adventitial lymphatic capillary expansion impacts on plaque T cell accumulation in atherosclerosis. Sci Rep 7:45263. 10.1038/srep45263 10.1038/srep45263 PMC5368662 28349940

[41] Lim HY, Thiam CH, Yeo KP, Bisoendial R, Hii CS, McGrath KC, Tan KW, Heather A, Alexander JS, Angeli V (2013) Lymphatic vessels are essential for the removal of cholesterol from peripheral tissues by SR-BI-mediated transport of HDL. Cell Metab 17:671–684. 10.1016/j.cmet.2013.04.002 10.1016/j.cmet.2013.04.002 23663736

[42] Milasan A, Smaani A, Martel C (2019) Early rescue of lymphatic function limits atherosclerosis progression in Ldlr-/- mice. Atherosclerosis 283:106–119. 10.1016/j.atherosclerosis.2019.01.031 10.1016/j.atherosclerosis.2019.01.031 30851674

[43] Martel C, Li W, Fulp B, Platt AM, Gautier EL, Westerterp M, Bittman R, Tall AR, Chen SH, Thomas MJ, Kreisel D, Swartz MA, Sorci-Thomas MG, Randolph GJ (2013) Lymphatic vasculature mediates macrophage reverse cholesterol transport in mice. J Clin Invest 123:1571–1579. 10.1172/JCI63685 10.1172/JCI63685 PMC3613904 23524964

[44] Gracia G, Cao E, Johnston APR, Porter CJH, Trevaskis NL (2020) Organ-specific lymphatics play distinct roles in regulating HDL trafficking and composition. Am J Physiol Gastrointest Liver Physiol 318:G725-G735. 10.1152/ajpgi.00340.2019 10.1152/ajpgi.00340.2019 32068443

[45] Out R, Hoekstra M, Habets K, Meurs I, de Waard V, Hildebrand RB, Wang Y, Chimini G, Kuiper J, Van Berkel TJ, Van Eck M (2008) Combined deletion of macrophage ABCA1 and ABCG1 leads to massive lipid accumulation in tissue macrophages and distinct atherosclerosis at relatively low plasma cholesterol levels. Arterioscler Thromb Vasc Biol 28:258–264. 10.1161/ATVBAHA.107.156935 10.1161/ATVBAHA.107.156935 18006857

[46] Trevaskis NL, Kaminskas LM, Porter CJ (2015) From sewer to saviour – targeting the lymphatic system to promote drug exposure and activity. Nat Rev Drug Discov 14:781–803. 10.1038/nrd4608 10.1038/nrd4608 26471369

[47] Tessier N, Moawad F, Amri N, Brambilla D, Martel C (2021) Focus on the lymphatic route to optimize drug delivery in cardiovascular medicine. Pharmaceutics 13:1200. 10.3390/pharmaceutics13081200 10.3390/pharmaceutics13081200 PMC8398144 34452161

[48] Weber C, Zernecke A, Libby P (2008) The multifaceted contributions of leukocyte subsets to atherosclerosis: lessons from mouse models. Nat Rev Immunol 8:802–815. 10.1038/nri2415 10.1038/nri2415 18825131

[49] Xing L, Ji RC (2008) Lymphangiogenesis, myeloid cells and inflammation. Expert Rev Clin Immunol 4:599–613. 10.1586/1744666X.4.5.599 10.1586/1744666X.4.5.599 20476963

[50] Hansson GK, Hermansson A (2011) The immune system in atherosclerosis. Nat Immunol 12:204–212. 10.1038/ni.2001 10.1038/ni.2001 21321594

[51] Kataru RP, Kim H, Jang C, Choi DK, Koh BI, Kim M, Gollamudi S, Kim YK, Lee SH, Koh GY (2011) T lymphocytes negatively regulate lymph node lymphatic vessel formation. Immunity 34:96–107. 10.1016/j.immuni.2010.12.016 10.1016/j.immuni.2010.12.016 21256057

[52] Ji RC, Eshita Y (2014) Rapamycin inhibition of CFA-induced lymphangiogenesis in PLN is independent of mast cells. Mol Biol Rep 41:2217–2228. 10.1007/s11033-014-3073-1 10.1007/s11033-014-3073-1 24420861

[53] Brakenhielm E, Alitalo K (2019) Cardiac lymphatics in health and disease. Nat Rev Cardiol 16:56–68. 10.1038/s41569-018-0087-8 10.1038/s41569-018-0087-8 30333526

[54] Duewell P, Kono H, Rayner KJ, Sirois CM, Vladimer G, Bauernfeind FG, Abela GS, Franchi L, Nuñez G, Schnurr M, Espevik T, Lien E, Fitzgerald KA, Rock KL, Moore KJ, Wright SD, Hornung V, Latz E (2010) NLRP3 inflammasomes are required for atherogenesis and activated by cholesterol crystals. Nature 464:1357–1361. 10.1038/nature08938 10.1038/nature08938 PMC2946640 20428172

[55] Hermansson A, Ketelhuth DF, Strodthoff D, Wurm M, Hansson EM, Nicoletti A, Paulsson-Berne G, Hansson GK (2010) Inhibition of T cell response to native low-density lipoprotein reduces atherosclerosis. J Exp Med 207:1081–1093. 10.1084/jem.20092243 10.1084/jem.20092243 PMC2867279 20439543

[56] Aspelund A, Robciuc MR, Karaman S, Makinen T, Alitalo K (2016) Lymphatic system in cardiovascular medicine. Circ Res 118:515–530. 10.1161/CIRCRESAHA.115.306544 10.1161/CIRCRESAHA.115.306544 26846644

[57] Singla B, Lin HP, Ahn W, White J, Csányi G (2021) Oxidatively modified LDL suppresses lymphangiogenesis via CD36 signaling. Antioxidants (Basel) 10:331. 10.3390/antiox10020331 10.3390/antiox10020331 PMC7926875 33672291

[58] Yeo KP, Lim HY, Thiam CH, Azhar SH, Tan C, Tang Y, See WQ, Koh XH, Zhao MH, Phua ML, Balachander A, Tan Y, Lim SY, Chew HS, Ng LG, Angeli V (2020) Efficient aortic lymphatic drainage is necessary for atherosclerosis regression induced by ezetimibe. Sci Adv 6:eabc2697. 10.1126/sciadv.abc2697 10.1126/sciadv.abc2697 PMC7732200 33310846

[59] Singla B, Lin HP, Chen A, Ahn W, Ghoshal P, Cherian-Shaw M, White J, Stansfield BK, Csányi G (2021) Role of R-spondin 2 in arterial lymphangiogenesis and atherosclerosis. Cardiovasc Res 117:1489–1509. 10.1093/cvr/cvaa244 10.1093/cvr/cvaa244 PMC8152716 32750106

[60] Agmon Y, Khandheria BK, Meissner I, Schwartz GL, Petterson TM, O'Fallon WM, Gentile F, Whisnant JP, Wiebers DO, Seward JB (2000) Independent association of high blood pressure and aortic atherosclerosis: a population-based study. Circulation 102:2087–2093. 10.1161/01.cir.102.17.2087 10.1161/01.cir.102.17.2087 11044425

[61] Machnik A, Neuhofer W, Jantsch J, Dahlmann A, Tammela T, Machura K, Park JK, Beck FX, Müller DN, Derer W, Goss J, Ziomber A, Dietsch P, Wagner H, van Rooijen N, Kurtz A, Hilgers KF, Alitalo K, Eckardt KU, Luft FC, Kerjaschki D, Titze J (2009) Macrophages regulate salt-dependent volume and blood pressure by a vascular endothelial growth factor-C-dependent buffering mechanism. Nat Med 15:545–552. 10.1038/nm.1960 10.1038/nm.1960 19412173

[62] Lopez Gelston CA, Balasubbramanian D, Abouelkheir GR, Lopez AH, Hudson KR, Johnson ER, Muthuchamy M, Mitchell BM, Rutkowski JM (2018) Enhancing renal lymphatic expansion prevents hypertension in mice. Circ Res 122:1094–1101. 10.1161/CIRCRESAHA.118.312765 10.1161/CIRCRESAHA.118.312765 29475981

[63] Balasubbramanian D, Gelston CAL, Lopez AH, Iskander G, Tate W, Holderness H, Rutkowski JM, Mitchell BM (2020) Augmenting renal lymphatic density prevents angiotensin II-induced hypertension in male and female mice. Am J Hypertens 33:61–69. 10.1093/ajh/hpz139 10.1093/ajh/hpz139 PMC6931897 31429865

[64] Beaini S, Saliba Y, Hajal J, Smayra V, Bakhos JJ, Joubran N, Chelala D, Fares N (2019) VEGF-C attenuates renal damage in salt-sensitive hypertension. J Cell Physiol 234:9616–9630. 10.1002/jcp.27648 10.1002/jcp.27648 30378108

[65] Song L, Chen X, Swanson TA, LaViolette B, Pang J, Cunio T, Nagle MW, Asano S, Hales K, Shipstone A, Sobon H, Al-Harthy SD, Ahn Y, Kreuser S, Robertson A, Ritenour C, Voigt F, Boucher M, Sun F, Sessa WC, Roth Flach RJ (2020) Lymphangiogenic therapy prevents cardiac dysfunction by ameliorating inflammation and hypertension. Elife 9:e58376. 10.7554/eLife.58376 10.7554/eLife.58376 PMC7695461 33200983

[66] Donnan MD, Kenig-Kozlovsky Y, Quaggin SE (2021) The lymphatics in kidney health and disease. Nat Rev Nephrol 17:655–675. 10.1038/s41581-021-00438-y 10.1038/s41581-021-00438-y 34158633

[67] Townsend EA, Miller VM, Prakash YS (2012) Sex differences and sex steroids in lung health and disease. Endocr Rev 33:1–47. 10.1210/er.2010-0031 10.1210/er.2010-0031 PMC3365843 22240244

[68] Boese AC, Kim SC, Yin KJ, Lee JP, Hamblin MH (2017) Sex differences in vascular physiology and pathophysiology: estrogen and androgen signaling in health and disease. Am J Physiol Heart Circ Physiol 313:H524-H545. 10.1152/ajpheart.00217.2016 10.1152/ajpheart.00217.2016 PMC5625178 28626075

[69] Mahmoodzadeh S, Leber J, Zhang X, Jaisser F, Messaoudi S, Morano I, Furth PA, Dworatzek E, Regitz-Zagrosek V (2014) Cardiomyocyte-specific estrogen receptor alpha increases angiogenesis, lymphangiogenesis and reduces fibrosis in the female mouse heart post-myocardial infarction. J Cell Sci Ther 5:153. 10.4172/2157-7013.1000153 10.4172/2157-7013.1000153 PMC4070011 24977106

[70] Pollow DP, Uhrlaub J, Romero-Aleshire M, Sandberg K, Nikolich-Zugich J, Brooks HL, Hay M (2014) Sex differences in T-lymphocyte tissue infiltration and development of angiotensin II hypertension. Hypertension 64:384–390. 10.1161/HYPERTENSIONAHA.114.03581 10.1161/HYPERTENSIONAHA.114.03581 PMC4096042 24890822

[71] Trincot CE, Xu W, Zhang H, Kulikauskas MR, Caranasos TG, Jensen BC, Sabine A, Petrova TV, Caron KM (2019) Adrenomedullin induces cardiac lymphangiogenesis after myocardial infarction and regulates cardiac edema via connexin 43. Circ Res 124:101–113. 10.1161/CIRCRESAHA.118.313835 10.1161/CIRCRESAHA.118.313835 PMC6318063 30582443

[72] Frangogiannis NG (2015) Pathophysiology of myocardial infarction. Compr Physiol 5:1841–1875. 10.1002/cphy.c150006 10.1002/cphy.c150006 26426469

[73] Dongaonkar RM, Stewart RH, Geissler HJ, Laine GA (2010) Myocardial microvascular permeability, interstitial oedema, and compromised cardiac function. Cardiovasc Res 87:331–339. 10.1093/cvr/cvq145 10.1093/cvr/cvq145 PMC2895547 20472566

[74] Nahrendorf M, Swirski FK, Aikawa E, Stangenberg L, Wurdinger T, Figueiredo JL, Libby P, Weissleder R, Pittet MJ (2007) The healing myocardium sequentially mobilizes two monocyte subsets with divergent and complementary functions. J Exp Med 204:3037–3047. 10.1084/jem.20070885 10.1084/jem.20070885 PMC2118517 18025128

[75] Dieterich LC, Seidel CD, Detmar M (2014) Lymphatic vessels: new targets for the treatment of inflammatory diseases. Angiogenesis 17:359–371. 10.1007/s10456-013-9406-1 10.1007/s10456-013-9406-1 24212981

[76] Vuorio T, Tirronen A, Ylä-Herttuala S (2017) Cardiac lymphatics - a new avenue for therapeutics? Trends Endocrinol Metab 28:285–296. 10.1016/j.tem.2016.12.002 10.1016/j.tem.2016.12.002 28087126

[77] Chen XG, Lv YX, Zhao D, Zhang L, Zheng F, Yang JY, Li XL, Wang L, Guo LY, Pan YM, Yan YW, Chen SY, Wang JN, Tang JM, Wan Y (2016) Vascular endothelial growth factor-C protects heart from ischemia/reperfusion injury by inhibiting cardiomyocyte apoptosis. Mol Cell Biochem 413:9–23. 10.1007/s11010-015-2622-9 10.1007/s11010-015-2622-9 26769665

[78] Lin QY, Zhang YL, Bai J, Liu JQ, Li HH (2021) VEGF-C/VEGFR-3 axis protects against pressure-overload induced cardiac dysfunction through regulation of lymphangiogenesis. Clin Transl Med 11:e374. 10.1002/ctm2.374 10.1002/ctm2.374 PMC7989711 33783987

[79] Vieira JM, Norman S, Villa Del Campo C, Cahill TJ, Barnette DN, Gunadasa-Rohling M, Johnson LA, Greaves DR, Carr CA, Jackson DG, Riley PR (2018) The cardiac lymphatic system stimulates resolution of inflammation following myocardial infarction. J Clin Invest 128:3402–3412. 10.1172/JCI97192 10.1172/JCI97192 PMC6063482 29985167

[80] Dorn LE, Petrosino JM, Wright P, Accornero F (2018) CTGF/CCN2 is an autocrine regulator of cardiac fibrosis. J Mol Cell Cardiol 121:205–211. 10.1016/j.yjmcc.2018.07.130 10.1016/j.yjmcc.2018.07.130 PMC6260782 30040954

[81] Ishikawa Y, Akishima-Fukasawa Y, Ito K, Akasaka Y, Tanaka M, Shimokawa R, Kimura-Matsumoto M, Morita H, Sato S, Kamata I, Ishii T (2007) Lymphangiogenesis in myocardial remodelling after infarction. Histopathology 51:345–353. 10.1111/j.1365-2559.2007.02785.x 10.1111/j.1365-2559.2007.02785.x PMC2366023 17727476

[82] Shimizu Y, Polavarapu R, Eskla KL, Pantner Y, Nicholson CK, Ishii M, Brunnhoelzl D, Mauria R, Husain A, Naqvi N, Murohara T, Calvert JW (2018)Impact of lymphangiogenesis on cardiac remodeling after ischemia and reperfusion injury. J Am Heart Assoc 7:e009565. 10.1161/JAHA.118.009565 10.1161/JAHA.118.009565 PMC6404883 30371303

[83] Maruyama K, Naemura K, Arima Y, Uchijima Y, Nagao H, Yoshihara K, Singh MK, Uemura A, Matsuzaki F, Yoshida Y, Kurihara Y, Miyagawa-Tomita S, Kurihara H (2021) Semaphorin3E-PlexinD1 signaling in coronary artery and lymphatic vessel development with clinical implications in myocardial recovery. iScience 24:102305. 10.1016/j.isci.2021.102305 10.1016/j.isci.2021.102305 PMC8041864 33870127

[84] Liao S, Cheng G, Conner DA, Huang Y, Kucherlapati RS, Munn LL, Ruddle NH, Jain RK, Fukumura D, Padera TP (2011) Impaired lymphatic contraction associated with immunosuppression. Proc Natl Acad Sci U S A 108:18784–18789. 10.1073/pnas.1116152108 10.1073/pnas.1116152108 PMC3219138 22065738

[85] Scallan JP, Zawieja SD, Castorena-Gonzalez JA, Davis MJ (2016) Lymphatic pumping: mechanics, mechanisms and malfunction. J Physiol 594:5749–5768. 10.1113/JP272088 10.1113/JP272088 PMC5063934 27219461

[86] Angeli V, Ginhoux F, Llodrà J, Quemeneur L, Frenette PS, Skobe M, Jessberger R, Merad M, Randolph GJ (2006) B cell-driven lymphangiogenesis in inflamed lymph nodes enhances dendritic cell mobilization. Immunity 24:203–215. 10.1016/j.immuni.2006.01.003 10.1016/j.immuni.2006.01.003 16473832

[87] Ji RC (2012) Macrophages are important mediators of either tumor- or inflammation-induced lymphangiogenesis. Cell Mol Life Sci 69:897–914. 10.1007/s00018-011-0848-6 10.1007/s00018-011-0848-6 PMC11114502 21984600

[88] Swirski FK, Nahrendorf M (2013) Leukocyte behavior in atherosclerosis, myocardial infarction, and heart failure. Science 339:161–166. 10.1126/science.1230719 10.1126/science.1230719 PMC3891792 23307733

[89] Frangogiannis NG (2014) The inflammatory response in myocardial injury, repair, and remodelling. Nat Rev Cardiol 11:255–265. 10.1038/nrcardio.2014.28 10.1038/nrcardio.2014.28 PMC4407144 24663091

[90] Davis KL, Laine GA, Geissler HJ, Mehlhorn U, Brennan M, Allen SJ (2000). Effects of myocardial edema on the development of myocardial interstitial fibrosis. Microcirculation.

[91] Adapala RK, Kanugula AK, Paruchuri S, Chilian WM, Thodeti CK (2020) TRPV4 deletion protects heart from myocardial infarction-induced adverse remodeling via modulation of cardiac fibroblast differentiation. Basic Res Cardiol 115:14. 10.1007/s00395-020-0775-5 10.1007/s00395-020-0775-5 PMC7322630 31925567

[92] Silvestre-Roig C, Fridlender ZG, Glogauer M, Scapini P (2019) Neutrophil diversity in health and disease. Trends Immunol 40:565–583. 10.1016/j.it.2019.04.012 10.1016/j.it.2019.04.012 PMC7185435 31160207

[93] Beyer M, Mallmann MR, Xue J, Staratschek-Jox A, Vorholt D, Krebs W, Sommer D, Sander J, Mertens C, Nino-Castro A, Schmidt SV, Schultze JL (2012) High-resolution transcriptome of human macrophages. PLoS One 7:e45466. 10.1371/journal.pone.0045466 10.1371/journal.pone.0045466 PMC3448669 23029029

[94] Peet C, Ivetic A, Bromage DI, Shah AM (2020) Cardiac monocytes and macrophages after myocardial infarction. Cardiovasc Res 116:1101–1112. 10.1093/cvr/cvz336 10.1093/cvr/cvz336 PMC7177720 31841135

[95] Kologrivova I, Shtatolkina M, Suslova T, Ryabov V (2021) Cells of the immune system in cardiac remodeling: main players in resolution of inflammation and repair after myocardial infarction. Front Immunol 12:664457. 10.3389/fimmu.2021.664457 10.3389/fimmu.2021.664457 PMC8050340 33868315

[96] Kim H, Kataru RP, Koh GY (2012) Regulation and implications of inflammatory lymphangiogenesis. Trends Immunol 33:350–356. 10.1016/j.it.2012.03.006 10.1016/j.it.2012.03.006 22579522

[97] Van der Borght K, Scott CL, Nindl V, Bouché A, Martens L, Sichien D, Van Moorleghem J, Vanheerswynghels M, De Prijck S, Saeys Y, Ludewig B, Gillebert T, Guilliams M, Carmeliet P, Lambrecht BN (2017) Myocardial infarction primes autoreactive T cells through activation of dendritic cells. Cell Rep 18:3005–3017. 10.1016/j.celrep.2017.02.079 10.1016/j.celrep.2017.02.079 PMC5379012 28329691

[98] Liao YH, Cheng X (2006) Autoimmunity in myocardial infarction. Int J Cardiol 112:21–26. 10.1016/j.ijcard.2006.05.009 10.1016/j.ijcard.2006.05.009 16837084

[99] Boag SE, Das R, Shmeleva EV, Bagnall A, Egred M, Howard N, Bennaceur K, Zaman A, Keavney B, Spyridopoulos I (2015) T lymphocytes and fractalkine contribute to myocardial ischemia/reperfusion injury in patients. J Clin Invest 125:3063–3076. 10.1172/JCI80055 10.1172/JCI80055 PMC4563749 26168217

[100] Hofmann U, Frantz S (2016) Role of T-cells in myocardial infarction. Eur Heart J 37:873–879. 10.1093/eurheartj/ehv639 10.1093/eurheartj/ehv639 26646702

[101] Karadimou G, Gisterå A, Gallina AL, Caravaca AS, Centa M, Salagianni M, Andreakos E, Hansson GK, Malin S, Olofsson PS, Paulsson-Berne G (2020) Treatment with a Toll-like Receptor 7 ligand evokes protective immunity against atherosclerosis in hypercholesterolaemic mice. J Intern Med 288:321–334. 10.1111/joim.13085 10.1111/joim.13085 32410352

[102] Kutkut I, Meens MJ, McKee TA, Bochaton-Piallat ML, Kwak BR (2015) Lymphatic vessels: an emerging actor in atherosclerotic plaque development. Eur J Clin Invest 45:100–108. 10.1111/eci.12372 10.1111/eci.12372 25388153

[103] Burger F, Miteva K, Baptista D, Roth A, Fraga-Silva RA, Martel C, Stergiopulos N, Mach F, Brandt KJ (2021) Follicular regulatory helper T cells control the response of regulatory B cells to a high-cholesterol diet. Cardiovasc Res 117:743–755. 10.1093/cvr/cvaa069 10.1093/cvr/cvaa069 PMC7898950 32219371

[104] Gu W, Ni Z, Tan YQ, Deng J, Zhang SJ, Lv ZC, Wang XJ, Chen T, Zhang Z, Hu Y, Jing ZC, Xu Q (2019) Adventitial cell atlas of wt (Wild Type) and ApoE (apolipoprotein E)-deficient mice defined by single-cell RNA sequencing. Arterioscler Thromb Vasc Biol 39:1055–1071. 10.1161/ATVBAHA.119.312399 10.1161/ATVBAHA.119.312399 PMC6553510 30943771

[105] Tadayon S, Dunkel J, Takeda A, Eichin D, Virtakoivu R, Elima K, Jalkanen S, Hollmén M (2021) Lymphatic endothelial cell activation and dendritic cell transmigration is modified by genetic deletion of Clever-1. Front Immunol 12:602122. 10.3389/fimmu.2021.602122. 10.3389/fimmu.2021.602122 PMC7970002 33746947

[106] Milasan A, Dallaire F, Mayer G, Martel C (2016) Effects of LDL receptor modulation on lymphatic function. Sci Rep 6:27862. 10.1038/srep27862 10.1038/srep27862 PMC4899717 27279328

[107] Loo CP, Nelson NA, Lane RS, Booth JL, Loprinzi Hardin SC, Thomas A, Slifka MK, Nolz JC, Lund AW (2017) Lymphatic vessels balance viral dissemination and immune activation following cutaneous viral infection. Cell Rep 20:3176–3187. 10.1016/j.celrep.2017.09.006. 10.1016/j.celrep.2017.09.006 PMC5621787 28954233

[108] Edwards LA, Nowocin AK, Jafari NV, Meader LL, Brown K, Sarde A, Lam C, Murray A, Wong W (2018) Chronic rejection of cardiac allografts is associated with increased lymphatic flow and cellular trafficking. Circulation 137:488–503. 10.1161/CIRCULATIONAHA.117.028533 10.1161/CIRCULATIONAHA.117.028533 PMC5737875 28775077

[109] Stacker SA, Achen MG (2018) Emerging roles for VEGF-D in human disease. Biomolecules 8:1. 10.3390/biom8010001 10.3390/biom8010001 PMC5871970 29300337

[110] Cursiefen C, Cao J, Chen L, Liu Y, Maruyama K, Jackson D, Kruse FE, Wiegand SJ, Dana MR, Streilein JW (2004) Inhibition of hemangiogenesis and lymphangiogenesis after normal-risk corneal transplantation by neutralizing VEGF promotes graft survival. Invest Ophthalmol Vis Sci 45:2666–2673. 10.1167/iovs.03-1380 10.1167/iovs.03-1380 15277490

[111] Jones D, Min W (2011) An overview of lymphatic vessels and their emerging role in cardiovascular disease. J Cardiovasc Dis Res 2:141–152. 10.4103/0975-3583.85260 10.4103/0975-3583.85260 PMC3195192 22022141

[112] Jonigk D, Lehmann U, Stuht S, Wilhelmi M, Haverich A, Kreipe H, Mengel M (2007) Recipient-derived neoangiogenesis of arterioles and lymphatics in quilty lesions of cardiac allografts. Transplantation 84:1335–1342. 10.1097/01.tp.0000287458.72440.75 10.1097/01.tp.0000287458.72440.75 18049119

[113] Brown K, Badar A, Sunassee K, Fernandes MA, Shariff H, Jurcevic S, Blower PJ, Sacks SH, Mullen GE, Wong W (2011) SPECT/CT lymphoscintigraphy of heterotopic cardiac grafts reveals novel sites of lymphatic drainage and T cell priming. Am J Transplant 11:225–234. 10.1111/j.1600-6143.2010.03388.x 10.1111/j.1600-6143.2010.03388.x PMC6211618 21219574

[114] Daly KP, Seifert ME, Chandraker A, Zurakowski D, Nohria A, Givertz MM, Karumanchi SA, Briscoe DM (2013) VEGF-C, VEGF-A and related angiogenesis factors as biomarkers of allograft vasculopathy in cardiac transplant recipients. J Heart Lung Transplant 32:120–128. 10.1016/j.healun.2012.09.030 10.1016/j.healun.2012.09.030 PMC3597743 23260712

[115] Kerjaschki D, Regele HM, Moosberger I, Nagy-Bojarski K, Watschinger B, Soleiman A, Birner P, Krieger S, Hovorka A, Silberhumer G, Laakkonen P, Petrova T, Langer B, Raab I (2004) Lymphatic neoangiogenesis in human kidney transplants is associated with immunologically active lymphocytic infiltrates. J Am Soc Nephrol 15:603–612. 10.1097/01.asn.0000113316.52371.2e 10.1097/01.asn.0000113316.52371.2e 14978162

[116] Stehlik J, Armstrong B, Baran DA, Bridges ND, Chandraker A, Gordon R, De Marco T, Givertz MM, Heroux A, Iklé D, Hunt J, Kfoury AG, Madsen JC, Morrison Y, Feller E, Pinney S, Tripathi S, Heeger PS, Starling RC (2019) Early immune biomarkers and intermediate-term outcomes after heart transplantation: results of Clinical Trials in Organ Transplantation-18. Am J Transplant 19:1518–1528. 10.1111/ajt.15218 10.1111/ajt.15218 PMC6482086 30549425

[117] Krebs R, Tikkanen JM, Ropponen JO, Jeltsch M, Jokinen JJ, Ylä-Herttuala S, Nykänen AI, Lemström KB (2012) Critical role of VEGF-C/VEGFR-3 signaling in innate and adaptive immune responses in experimental obliterative bronchiolitis. Am J Pathol 181:1607–1620. 10.1016/j.ajpath.2012.07.021 10.1016/j.ajpath.2012.07.021 22959907

[118] Emami-Naeini P, Dohlman TH, Omoto M, Hattori T, Chen Y, Lee HS, Chauhan SK, Dana R (2014) Soluble vascular endothelial growth factor receptor-3 suppresses allosensitization and promotes corneal allograft survival. Graefes Arch Clin Exp Ophthalmol 252:1755–1762. 10.1007/s00417-014-2749-5 10.1007/s00417-014-2749-5 PMC4221529 25091513

[119] Yin N, Zhang N, Xu J, Shi Q, Ding Y, Bromberg JS (2011) Targeting lymphangiogenesis after islet transplantation prolongs islet allograft survival. Transplantation 92:25–30. 10.1097/TP.0b013e31821d2661 10.1097/TP.0b013e31821d2661 PMC3703312 21508896

[120] Hos D, Matthaei M, Bock F, Maruyama K, Notara M, Clahsen T, Hou Y, Le VNH, Salabarria AC, Horstmann J, Bachmann BO, Cursiefen C (2019) Immune reactions after modern lamellar (DALK, DSAEK, DMEK) versus conventional penetrating corneal transplantation. Prog Retin Eye Res 73:100768. 10.1016/j.preteyeres.2019.07.001 10.1016/j.preteyeres.2019.07.001 31279005

[121] Podgrabinska S, Kamalu O, Mayer L, Shimaoka M, Snoeck H, Randolph GJ, Skobe M (2009) Inflamed lymphatic endothelium suppresses dendritic cell maturation and function via Mac-1/ICAM-1-dependent mechanism. J Immunol 183:1767–1779. 10.4049/jimmunol.0802167 10.4049/jimmunol.0802167 PMC4410990 19587009

[122] Card CM, Yu SS, Swartz MA (2014) Emerging roles of lymphatic endothelium in regulating adaptive immunity. J Clin Invest 124:943–952. 10.1172/JCI73316 10.1172/JCI73316 PMC3938271 24590280

[123] Dohlman TH, Omoto M, Hua J, Stevenson W, Lee SM, Chauhan SK, Dana R (2015) VEGF-trap aflibercept significantly improves long-term graft survival in high-risk corneal transplantation. Transplantation 99:678–686. 10.1097/TP.0000000000000512 10.1097/TP.0000000000000512 25606789

[124] Azzi J, Yin Q, Uehara M, Ohori S, Tang L, Cai K, Ichimura T, McGrath M, Maarouf O, Kefaloyianni E, Loughhead S, Petr J, Sun Q, Kwon M, Tullius S, von Andrian UH, Cheng J, Abdi R (2016) Targeted delivery of immunomodulators to lymph nodes. Cell Rep 15:1202–1213. 10.1016/j.celrep.2016.04.007 10.1016/j.celrep.2016.04.007 PMC4973867 27134176

[125] Yamagami S, Dana MR, Tsuru T (2002) Draining lymph nodes play an essential role in alloimmunity generated in response to high-risk corneal transplantation. Cornea 21:405–409. 10.1097/00003226-200205000-00014 10.1097/00003226-200205000-00014 11973391

[126] Li W, Gauthier JM, Tong AY, Terada Y, Higashikubo R, Frye CC, Harrison MS, Hashimoto K, Bery AI, Ritter JH, Nava RG, Puri V, Wong BW, Lavine KJ, Bharat A, Krupnick AS, Gelman AE, Kreisel D (2020) Lymphatic drainage from bronchus-associated lymphoid tissue in tolerant lung allografts promotes peripheral tolerance. J Clin Invest 130:6718–6727. 10.1172/JCI136057 10.1172/JCI136057 PMC7685742 33196461

[127] Cui Y, Liu K, Monzon-Medina ME, Padera RF, Wang H, George G, Toprak D, Abdelnour E, D'Agostino E, Goldberg HJ, Perrella MA, Forteza RM, Rosas IO, Visner G, El-Chemaly S (2015) Therapeutic lymphangiogenesis ameliorates established acute lung allograft rejection. J Clin Invest 125:4255–4268. 10.1172/JCI79693 10.1172/JCI79693 PMC4639995 26485284

[128] Nielsen NR, Rangarajan KV, Mao L, Rockman HA, Caron KM (2020) A murine model of increased coronary sinus pressure induces myocardial edema with cardiac lymphatic dilation and fibrosis. Am J Physiol Heart Circ Physiol 318:H895-H907. 10.1152/ajpheart.00436.2019 10.1152/ajpheart.00436.2019 PMC7191489 32142379

[129] Berta J, Hoda MA, Laszlo V, Rozsas A, Garay T, Torok S, Grusch M, Berger W, Paku S, Renyi-Vamos F, Masri B, Tovari J, Groger M, Klepetko W, Hegedus B, Dome B (2014) Apelin promotes lymphangiogenesis and lymph node metastasis. Oncotarget 5:4426–4437. 10.18632/oncotarget.2032 10.18632/oncotarget.2032 PMC4147335 24962866

[130] Ashley EA, Powers J, Chen M, Kundu R, Finsterbach T, Caffarelli A, Deng A, Eichhorn J, Mahajan R, Agrawal R, Greve J, Robbins R, Patterson AJ, Bernstein D, Quertermous T (2005) The endogenous peptide apelin potently improves cardiac contractility and reduces cardiac loading in vivo. Cardiovasc Res 65:73–82. 10.1016/j.cardiores.2004.08.018 10.1016/j.cardiores.2004.08.018 PMC2517138 15621035

[131] Caron KM, Smithies O (2001) Extreme hydrops fetalis and cardiovascular abnormalities in mice lacking a functional Adrenomedullin gene. Proc Natl Acad Sci U S A 98:615–619. 10.1073/pnas.021548898 10.1073/pnas.021548898 PMC14636 11149956

[132] Zhang HF, Wang YL, Tan YZ, Wang HJ, Tao P, Zhou P (2019) Enhancement of cardiac lymphangiogenesis by transplantation of CD34(+)VEGFR-3(+) endothelial progenitor cells and sustained release of VEGF-C. Basic Res Cardiol 114:43. 10.1007/s00395-019-0752-z 10.1007/s00395-019-0752-z PMC6778587 31587086

[133] Wang YL, Yu SN, Shen HR, Wang HJ, Wu XP, Wang QL, Zhou B, Tan YZ (2021) Thymosin β4 released from functionalized self-assembling peptide activates epicardium and enhances repair of infarcted myocardium. Theranostics 11:4262–4280. 10.7150/thno.52309. eCollection 2021 10.7150/thno.52309 PMC7977468 33754060

[134] Liu X, Pasula S, Song H, Tessneer KL, Dong Y, Hahn S, Yago T, Brophy ML, Chang B, Cai X, Wu H, McManus J, Ichise H, Georgescu C, Wren JD, Griffin C, Xia L, Srinivasan RS, Chen H (2014) Temporal and spatial regulation of epsin abundance and VEGFR3 signaling are required for lymphatic valve formation and function. Sci Signal 7:ra97. 10.1126/scisignal.2005413 10.1126/scisignal.2005413 PMC4226761 25314967

[135] Wu H, Rahman HNA, Dong Y, Liu X, Lee Y, Wen A, To KH, Xiao L, Birsner AE, Bazinet L, Wong S, Song K, Brophy ML, Mahamud MR, Chang B, Cai X, Pasula S, Kwak S, Yang W, Bischoff J, Xu J, Bielenberg DR, Dixon JB, D'Amato RJ, Srinivasan RS, Chen H (2018) Epsin deficiency promotes lymphangiogenesis through regulation of VEGFR3 degradation in diabetes. J Clin Invest 128:4025–4043. 10.1172/JCI96063 10.1172/JCI96063 PMC6118634 30102256

[136] Korpela H, Järveläinen N, Siimes S, Lampela J, Airaksinen J, Valli K, Turunen M, Pajula J, Nurro J, Ylä-Herttuala S (2021) Gene therapy for ischaemic heart disease and heart failure. J Intern Med. 10.1111/joim.13308 Online ahead of print 10.1111/joim.13308 34033164

